# Primary care biomarkers and dementia in people of the Torres Strait, Australia: extended data analysis

**DOI:** 10.3389/frdem.2023.1218709

**Published:** 2023-07-31

**Authors:** Fintan Thompson, Sarah Russell, Rachel Quigley, Malcolm McDonald, Betty Sagigi, Sean Taylor, Sandy Campbell, Barbara Schmidt, Adrian Esterman, Linton R. Harriss, Gavin Miller, Phillip Mills, Edward Strivens, Robyn McDermott

**Affiliations:** ^1^Australian Institute of Tropical Health and Medicine, College of Public Health, Medical and Veterinary Sciences, James Cook University, Cairns, QLD, Australia; ^2^School of Health Sciences, University of South Australia, Adelaide, SA, Australia; ^3^College of Medicine and Dentistry, James Cook University, Cairns, QLD, Australia; ^4^Queensland Health, Cairns and Hinterland Hospital and Health Service, Cairns, QLD, Australia; ^5^Queensland Health, Torres and Cape Hospital and Health Service, Thursday Island, QLD, Australia; ^6^Top End Health Service, Northern Territory Government, Darwin, NT, Australia; ^7^Molly Wardaguga Research Centre, Charles Darwin University, Brisbane, QLD, Australia

**Keywords:** dementia, prevention, First Nations, Indigenous, Australia

## Abstract

**Objective:**

Dementia disproportionately affects First Nations populations. Biomarkers collected in primary care may assist with determining dementia risk. Our previous underpowered study showed some suggestive associations between baseline biomarkers with follow-up dementia or cognitive impairment. The current study extended this work with a larger linked dataset.

**Study design and setting:**

Probabilistic data linkage was used to combine four baseline datasets with one follow-up assessment of dementia status 0–20 years later in a First Nations population in Australia. Mixed Effects Generalized Linear Regression models were used to test associations between baseline measures and follow-up status, accounting for repeated measures within individuals.

**Results:**

Linked data were available for 88 individuals, with 101–279 baseline observations, depending on the type of measure. Higher urinary albumin to creatine ratio was associated with greater risk of cognitive impairment/dementia, whereas body weight and key lipid markers were negatively associated. There was no clear trend when these associations were examined by timing of measurement (i.e., ≤10 years or >10 years before a dementia assessment).

**Conclusions:**

The results of this study support findings from our previous work and indicate that microalbuminuria can be an early indicator of dementia risk in this population. The weight and lipid profile findings reflect the mixed results in the published literature and require further investigation and interpretation.

## 1. Introduction

The First Nations peoples of Australia, Aboriginal peoples and Torres Strait Islanders, are among the oldest continuous populations in the world (Rasmussen et al., 2011). Through their resilience, ingenuity, land management, and social systems, this population thrived for over sixty thousand years before foreign occupation. Colonization continues to create social and health inequalities, but improvements are evident in certain indicators, including reduced smoking rates (Australian Institute of Health Welfare, 2022), and increased school enrolment (Australian Bureau of Statistics, 2020). In addition, First Nations-led responses to specific acute health issues, such as the COVID-19 pandemic (Crooks et al., 2020), have been innovative and timely, reflecting the ongoing resilience of this population.

Dementia rates are higher in First Nations Australians, including people of the Torres Strait, compared to the wider Australian population (Smith et al., 2008; Radford et al., 2015; Russell S. G. et al., 2021). This disparity likely reflects increased life-course exposure to modifiable dementia risks, which is due to upstream socioeconomic determinants (Walker et al., 2020). Consequently, a substantial proportion of dementia in First Nations Australians may be potentially preventable (Thompson et al., 2022a; Sue See et al., 2023). Given that dyslipidaemia and chronic kidney disease (CKD) are common conditions for First Nations Australians, the indicators accompanying these conditions may provide avenues for early identification of dementia risk. Our previous data linkage study of dementia risks in First Nations Australians showed modest suggestive but mixed associations between baseline kidney dysfunction, body weight and lipid measures with follow-up dementia or cognitive impairment (Thompson et al., 2022b). However, small numbers and a restricted time-period of data collection (i.e., 10–20 years) limited the conclusions.

The link between microalbuminuria, as measured by the urine albumin/creatinine ratio (UACR), subsequent kidney disease and cardiovascular disease was established more than 20 years ago (Weir, 2007). One likely mechanism is that a raised UACR flags endothelial dysfunction and reflects subclinical vascular damage in the kidneys and other vascular beds (Ekblad et al., 2018). It has become increasingly clear that microalbuminuria is also an early indicator of later cognitive health and dementia (Vupputuri et al., 2008; Georgakis et al., 2017). As such, UACR could become a useful clinical tool in primary health for determining dementia risk, especially for First Nations Australians.

Conventional wisdom has been that obesity, high plasma triglycerides and dyslipidemia are risk factors for Alzheimer's disease and all-cause dementia (Whitmer et al., 2005; Iwagami et al., 2021; Nordestgaard et al., 2021). However, the true picture is more complicated, and large studies have shown opposite findings (Panza et al., 2006). As such, the preventative role of statins in dementia has yet to be proven (Lee et al., 2019; Lee Y.-B. et al., 2022; Olmastroni et al., 2022). It could be that the direction of association depends on the timing of weight and lipid measurement relative to a person's age and dementia clinical onset. Dyslipidaemia in midlife may contribute to later pathogenesis of dementia, whereas lower lipid levels in later life may reflect systemic inflammatory and lifestyle changes that accompany the course of preclinical dementia. Long-term prospective studies are needed to better understand these associations and causations. The apparent contradictions in this field leave the clinical role of lipid lowering medication in the prevention of dementia unresolved.

The current study aimed to extend our previous work by using a larger linked dataset, with repeated measures for individuals over a longer baseline time period (i.e., 0–20 years). This study sought to examine associations between health measures collected in midlife and later life, with dementia status data collected in later life. The objectives were to: (1) link datasets from multiple research projects to create a single longitudinal dataset for an Australian First Nations population; (2) test associations between baseline pathology measures collected in primary care and follow-up dementia status; and (3) examine how the number of years elapsed between baseline measures and dementia assessment affected the strength and direction of these associations.

## 2. Materials and methods

### 2.1. Study design and population

This retrospective cohort study linked four baseline datasets to one “follow-up” dataset, to create a single longitudinal dataset. The final combined dataset comprised repeated baseline health measures for a cohort of individuals and their outcome diagnosis from a dementia assessment at follow-up. While all projects were conducted independently and at different time points, they occurred in the same geographic regions and populations. The baseline projects were the Well Person's Health Check (WPHC) (Miller et al., 2002), Getting Better at Chronic Care (GBACC) (Schmidt et al., 2012), Primary Health Care Models (PHCM) (Taylor et al., 2016, 2017; Taylor, 2017), and the Zenadth-Kes Health Partnership (ZKHP) (Berger et al., 2019). The follow-up project was the Torres Strait Dementia Prevalence Survey (TSDPS) (Russell S. G. et al., 2021). The details of each study and the data linkage are summarized below. Ethics approval to link the datasets from these studies was provided by the Far North Queensland Human Research Ethics Committee (AM/2022/QCH/41243), and Public Health Act approval to link secondary data was granted by the Queensland Government Office of Research and Innovation (PHA 41243.1).

### 2.2. Baseline projects

The WPHC project (1998–2000) was a community screening program of 3,033 First Nations residents aged 13 years and over living in 26 rural and remote communities in northern Queensland. Health data were obtained directly from participants via self-report and pathology samples. An additional WPHC (2005–2007) followed up participants from the first check and recruited new participants. The GBACC project (2010–2015) was a cluster randomized controlled trial of intensive management of chronic conditions in 197 First Nations participants who attended rural and remote primary health care services in Far North Queensland. Data were obtained directly from participants and via primary healthcare record audits. The PHCM (2012–2015) was a retrospective clustered cohort study to assess the effectiveness of dedicated clinical support on diabetes mellitus risk factors in 285 First Nations residents living with type 2 diabetes mellitus (T2DM) in the Torres Strait. The data were obtained directly from participants and via primary healthcare record audits. The ZKHP (2016) was a community-based health-screening program of 210 First Nations residents aged 15–78 years residing on one inner island and one outer island of the Torres Strait. The aim was to explore the association between metabolic syndrome and other chronic health conditions. The data were obtained directly from participants via self-report and pathology samples.

### 2.3. Follow-up project

The TSDPS (2015–2018) was a cross-sectional survey of 274 First Nations residents of the Torres Strait region to determine the prevalence of dementia. Recruitment was limited to people aged ≥40 years. There were no other inclusion or exclusion criteria, as the aim was to provide a representative sample of the geographic region. The research team administered the Kimberley Indigenous Cognitive Assessment tool (KICA) to participants to collect self-reported clinical and functional information, which included a brief cognitive screen for dementia. Geriatricians in the research team also reviewed participants and examined disease status, physical health, and cognitive functioning and reviewed medical records. The geriatricians did not use KICA results to inform their medical assessments.

### 2.4. Data linkage and dataset structure

The primary author (FT) conducted probabilistic data linkage using the Stata 15 (StataCorp, 2017) package “*dtalink*” (Kranker, 2018). The unique participant identifiers in each dataset were names, dates of birth, and gender. The Stata “*calcweights*” command determined the weighting for each unique identifier. The data from the TSDPS were linked to each baseline research project over successive rounds until all potential linkages were identified. The primary author (FT) did a final clerical review of the linked data. All datasets were combined into a single long-format dataset, where observations for individuals were stored across multiple rows of data. Static baseline variables that did not change, comprising gender, highest education, and age at dementia diagnosis, were copied to each row of data. Other study variables, such as pathology and anthropometric measures, differed across time points. Supplementary material 1 provides a de-identified visual example of how one pathology measure (i.e., triglycerides) was collected for individual participants throughout the study period. Supplementary material 1 also provides a de-identified example structure of the data used for analyses. Supplementary Box 1 lists the manuscript and supplementary tables and figures in this study and provides a plain language description of the purpose of each.

### 2.5. Predictor variables—Description

Supplementary Figure 1 shows health measures that were comparable across the four baseline datasets. For the current study, measures available in at least three baseline datasets were selected for linkage and analyses. The exceptions were very low-density lipoprotein (VLDL), which provided an alternative measure of triglycerides, and highest educational attainment, sourced from the TSDPS via self-report. The baseline measures that were collected in at least three baseline studies were body weight (kilograms, kg), Body Mass Index [weight (kg)/height^2^, BMI], waist circumference (centimeters, cm), any alcohol consumption (Yes/No), and cigarette smoking (i.e., self-reported “currently smoked tobacco,” Yes/No). T2DM and hypertension status were assessed at the time of data collection and the method varied by baseline study (e.g., self-report, pathology measures, or medical records). Systolic and diastolic blood pressure were recorded as millimeters of mercury (mmHg). Pathology records were used to collect measures for hemoglobin A1c (HbA1c, %) and millimoles per liter (mmol/L) for triglycerides, total cholesterol, high-density lipoprotein, LDL, and VLDL. Urine samples were used for albumin creatinine ratio. Supplementary Table 1 shows the number of observations for each variable across the four baseline datasets.

Continuous outliers for the baseline measures, by dementia status, were identified using Quartiles 1 and 3 (i.e., Q1 and Q3) and the Interquartile Range (iqr). Values lower than Q1-(iqr^*^1.5) or greater than Q3 + (iqr^*^1.5) were considered outliers and winsorized by replacing these values with the next lowest or highest non-outlying value. Supplementary material 2 shows boxplots of continuous measures, by dementia status, before and after winsorizing. Supplementary Table 2 shows that most continuous variables had nine or fewer outliers, except for UACR, which had 29 outlying values. The main analyses in this study used the variables in three forms; outliers included (i.e., original measures), with outliers winsorized, and outliers removed. For UACR, additional analyses were also conducted after removing only the two most extreme outliers (i.e., >300). Supplementary material 2 shows the position of these outlying values relative to other measures of UACR.

The time elapsed between each baseline observation and the respective TSDPS assessment was calculated as the years between these measures (Supplementary Table 3). Eleven people participated in the ZKHP within 12 months after the TSDPS (i.e., within 12 months after the “end-point” measure). To include time elapsed in modeling, the dates for each observation from these 11 assessments was changed to the day before the TSDPS. To determine whether changing this date information impacted the results, sensitivity analyses were conducted by testing the study's main findings, excluding all ZKHP observations. For all data collections combined, date information was missing for measures of hypertension (*n* = 100), T2DM (*n* = 72), smoking (*n* = 45), BMI (*n* = 30), weight (*n* = 2), and alcohol (*n* = 1) (data not tabled). All other observations were accompanied by date information. The timing of collection of each baseline measure was separated into two categories from the time of TSDPS assessment. These time periods were within 10 years (i.e., 0–10.0 years) and greater than 10 years (Supplementary Table 4).

As VLDL was only available in the WPHC and ZKHP, this indicator had limited observations. For post-hoc analyses, an alternative VLDL measure was derived from TG using the formula TG^*^0.166, described in Wilson et al. (1985).

### 2.6. Follow-up/outcome variables

The primary outcome measure was cognitive status based on results of the TSDPS. A panel of geriatricians and an older person's psychiatrist blind-reviewed results from the comprehensive geriatrician assessments to provide consensus diagnoses based on the Diagnostic and Statistical Manual for Mental Disorders, 4th Edition (DSM-IV TR) criteria (Frances et al., 1995). Participants were then reclassified as normal cognition, dementia, or cognitive impairment no dementia (CIND) based on their diagnoses. The CIND group comprised people who met DSM IV-TR criteria of Cognitive Disorder-Not Otherwise Specified (NOS) or Amnestic Disorder-NOS, such as cognitive decline without significant impact on activities of daily living. The full details of this method have been published elsewhere (Russell S. et al., 2021). As there were a small number of participants with dementia at follow-up, people who had either CIND or dementia were combined into a single group for analyses. The final dichotomous outcome for the current study was therefore no cognitive impairment (i.e., normal cognition) or CIND/dementia. While dementia and CIND differ, both states are characterized by an objective decrease in cognitive functioning relative to age expectations and share similar etiologies. Supplementary Table 5 describes the characteristics, diagnoses, health behaviors, and pathology measures of participants who had their data linked in the current study.

### 2.7. Statistical analysis

All analyses were conducted using the Stata 15 software package (StataCorp, 2017), *p*-values <0.05 were considered statistically significant, and *p* < 0.10 as trend significant. Categorical variables were tested against the outcome measure using Pearson chi-square tests for independence or Fisher's Exact tests for expected cell counts <5 (Table 1). Continuous variables were assessed for normality and examined using mean and standard deviation (*SD*) when normally distributed or otherwise as median and iqr, with appropriate significance tests (e.g., Independent samples *t*-test or Kruskal-Wallis rank sum tests).

**Table 1:** Distribution of baseline variables by CIND/dementia status at follow-up for 88 individuals who participated in the Torres Strait Dementia Prevalence Survey (TSDPS) with at least one baseline assessment.

**Variables**		**Normal**			**CIND/Dementia**		**Tests**	
		***n***	**(%)**		***n***	**(%)**	**Chi** ^b^	***p***
**Follow up (Individuals)**		**55**	**(100.0)**		**33**	**(100.0)**		
**Gender**								
Male		17	(30.9)		7	(21.2)	0.98	0.323
Female		38	(69.1)		26	(78.8)		
**Education**								
Primary		11	(20.4)		16	(55.2)	10.99	0.004
Any high school		19	(35.2)		4	(13.8)		
Post school		24	(44.4)		9	(31.0)		
**Education**								
Grade school		30	(55.6)		20	(69.0)	1.42	0.234
Post school		24	(44.4)		9	(31.0)		
**Baseline (Observations)**		**(Total** **=** **sum of observations)**			**(Total** **=** **sum of observations)**			
**Smoking**								
No		118	(75.2)		77	(88.5)	6.21	0.013
Yes		39	(24.8)		10	(11.5)		
**Alcohol**								
No		57	(54.3)		34	(70.8)	3.74	0.053
Yes		48	(45.7)		14	(29.2)		
**Diabetes mellitus**								
No		44	(24.6)		16	(16.0)	2.80	0.094
Yes		135	(75.4)		84	(84.0)		
**Hypertension**								
No		48	(27.6)		12	(12.0)	9.02	0.003
Yes		126	(72.4)		88	(88.0)		
**Albuminuria**								
No		73	(61.9)		40	(52.6)	1.62	0.203
Yes		45	(38.1)		36	(47.4)		
	***n***	**Mean (sd)/Med (iqr)**		***n***	**Mean (sd)/Med (iqr)**		**RR/Chi** ^**2**^	***p***
**Original measures**								
Weight (kg)^a^	167	96.1	(21.8)	99	84.3	(16.4)	0.98	0.000
BMI^a^	152	34.7	(7.9)	89	32.0	(6.6)	0.97	0.005
Waist (cm)^a^	128	113.4	(15.5)	76	109.9	(14.6)	0.99	0.093
Systolic BP (mmHg)^a^	176	130.7	(15.6)	98	134.5	(19.5)	1.01	0.077
Diastolic BP (mmHg)^a^	176	74.1	(12.4)	98	73.5	(11.3)	1.00	0.711
HbA1c (% NGSP)^a^	137	8.2	(2.2)	79	8.0	(2.1)	0.98	0.577
UACR (ratio)^b^	118	1.4	(0.6–9.1)	76	2.7	(0.8–23.0)	4.88	0.027
Triglycerides (mmol/L)^b^	150	1.7	(1.1–2.3)	87	1.4	(0.9–2.0)	6.82	0.009
Cholesterol (mmol/L)^a^	166	4.7	(1.1)	97	4.3	(1.1)	0.83	0.025
HDL (mmol/L)^a^	166	1.1	(0.3)	97	1.1	(0.2)	1.37	0.285
LDL (mmol/L)^b^	162	2.7	(2.2–3.3)	90	2.2	(1.9–3.1)	7.79	0.005
VLDL (mmol/L)^b^	76	0.8	(0.5–1.1)	25	0.7	(0.4–0.8)	2.93	0.087
**Winsorized measures**								
Weight (kg)^a^(*n =* 7)	167	95.6	(20.5)	99	84.3	(16.4)	0.98	0.000
UACR (ratio)^a^ (*n =* 29)	118	1.4	(0.6–9.1)	76	2.7	(0.8–23.0)	7.03	0.008
Triglycerides (mmol/L)^a^ (*n =* 9)	150	1.7	(1.1–2.3)	87	1.4	(0.9–2.0)	7.26	0.007
Cholesterol (mmol/L)^a^ (*n =* 8)	166	4.7	(1.0)	97	4.3	(1.1)	0.82	0.023
LDL (mmol/L)^b^ (*n =* 5)	162	2.7	(2.2–3.3)	90	2.2	(1.9–3.1)	7.79	0.005
**Outliers removed**								
Weight (kg)^a^ (*n =* 7)	160	93.6	(18.5)	99	84.3	(16.4)	0.98	0.000
UACR (ratio)^b^ (*n =* 29)	100	0.9	(0.5–3.5)	65	1.9	(0.7–11.0)	7.20	0.007
UACR (ratio)^b^ (*n =* 2)	116	1.3	(0.6–7.6)	76	2.7	(0.8–23.0)	5.96	0.015
Triglycerides (mmol/L)^b^ (*n =* 9)	145	1.7	(1.1–2.2)	83	1.4	(0.9–1.8)	8.72	0.003
Cholesterol (mmol/L)^a^ (*n =* 8)	160	4.6	(0.9)	95	4.3	(1.0)	0.80	0.020
LDL (mmol/L)^b^ (*n =* 5)	157	2.7	(2.1–3.2)	90	2.2	(1.9–3.1)	5.99	0.014

CIND, Cognitive Impairment No Dementia; Weight, body weight (kilograms, kg); BMI, Body Mass Index [weight(kg)/height2]; Waist, waist circumference (centimeters, cm); Smoking, self-reported “currently smoked tobacco;” Alcohol, self-reported any alcohol consumption (Yes/No); Systolic BP, systolic blood pressure; Diastolic BP, diastolic blood pressure; HbA1c, hemoglobin A1c (%); UACR, Urinary albumin creatinine ratio; HDL, high-density lipoprotein; LDL, low-density lipoprotein; VLDL, very low-density lipoprotein.

^a^Normally distributed, differences tested with Independent samples t-test.

^b^Not normally distributed, differences tested with Kruskal-Wallis rank sum tests, Winsorized measures (n = number of observations winsorized), Outliers removed (n = number of observations removed).

To test the associations between repeat baseline measures and follow up CIND/dementia status while accounting for within person variation due to repeated estimates (i.e., clustering), relative risk (RR) estimates were derived using Mixed Effects Generalized Linear Modeling (*meglm*), with Poisson family type and robust error variance. In these analyses, CIND/dementia status was the dichotomized dependent variable (DV), and baseline measures were the independent variables (IV). Age was significantly associated with many baseline measures and was a prominent risk factor for CIND/dementia. To address this potential confounding, RR analyses were adjusted for age (i.e., aRR). Additional analyses also adjusted for the number of years between baseline observations and the follow-up dementia assessment. The Stata command for these analyses was: *meglmDVIV*_1_ *IV*_2_ *IV*_3_ *|| ID:, fam(pois) link(log) vce(robust)*, adjusted for age, and number of years (DV = dependent variable, IV_*n* =_ independent variable). Selected adjusted *meglm* results are presented in Table 2.

**Table 2:** Mixed Effects Generalized Linear Modeling (*meglm*) with relative risk (RR) associations between selected baseline measures and a diagnosis of Cognitive Impairment No Dementia (CIND) or dementia at follow-up for 88 people who participated in at least one baseline study, unadjusted and adjusted for age, original measures, winsorized outliers, and outliers removed.

**Variables**		**CIND or dementia**	**Adjusted—age**			**Adjusted—age, years**		
	***N***	**No/yes**	**RR**	**(95%CI)**	***p***	**RR**	**(95%CI)**	***p***
**Original measures**								
Weight (kg)	266	167/99	0.98	(0.97–1.00)	0.042	0.98	(0.97–1.00)	0.056
HbA1c (% NGSP)	216	137/79	1.09	(0.99–1.19)	0.085	1.08	(0.98–1.19)	0.111
UACR (ratio)	194	118/76	1.00	(1.00–1.00)	0.466	1.00	(1.00–1.00)	0.493
Triglycerides (mmol/L)	237	150/87	0.90	(0.71–1.14)	0.364	0.90	(0.70–1.16)	0.425
Cholesterol (mmol/L)	263	166/97	0.85	(0.70–1.03)	0.094	0.84	(0.68–1.04)	0.117
VLDL (mmol/L)	101	76/25	0.30	(0.09–1.01)	0.052	0.30	(0.09–0.99)	0.048
**Winsorized outliers**								
Weight (kg) (*n =* 7)	266	167/99	0.98	(0.97–1.00)	0.047	0.98	(0.97–1.00)	0.063
HbA1c (% NGSP) (*n =* 1)	216	137/79	1.09	(0.99–1.19)	0.082	1.08	(0.98–1.19)	0.107
UACR (ratio) (*n =* 29)	194	118/76	1.02	(1.01–1.03)	0.001	1.02	(1.01–1.03)	0.001
Cholesterol (mmol/L) (*n =* 8)	263	166/97	0.84	(0.69–1.03)	0.092	0.83	(0.67–1.05)	0.116
Triglycerides (mmol/L) (*n =* 9)	237	150/87	0.75	(0.55–1.03)	0.076	0.76	(0.55–1.04)	0.090
**Outliers removed**								
Weight (kg) (*n =* 7)	259	160/99	0.98	(0.97–1.00)	0.075	0.98	(0.97–1.00)	0.102
HbA1c (% NGSP) (*n =* 1)	215	136/79	1.09	(0.99–1.20)	0.075	1.09	(0.99–1.20)	0.097
UACR (ratio) (*n =* 29)	165	100/65	1.03	(1.01–1.04)	0.000	1.02	(1.01–1.03)	0.000
UACR (ratio) (*n =* 2)	192	116/76	1.00	(1.00–1.01)	0.003	1.00	(1.00–1.01)	0.003
Triglycerides (mmol/L) (*n =* 9)	228	145/83	0.69	(0.47–0.99)	0.045	0.68	(0.47–0.99)	0.045
Cholesterol (mmol/L) (*n =* 8)	255	160/95	0.82	(0.66–1.02)	0.079	0.82	(0.65–1.04)	0.104

Weight, body weight (kilograms, kg); UACR, Urinary albumin to creatinine ratio; LDL, low-density lipoprotein; VLDL, very low-density lipoprotein.

Winsorized measures (n = number of outlying observations winsorized); Outliers removed (n = number of outlying observations removed).

## 3. Results

### 3.1. Data linkage

**Figure 1:**
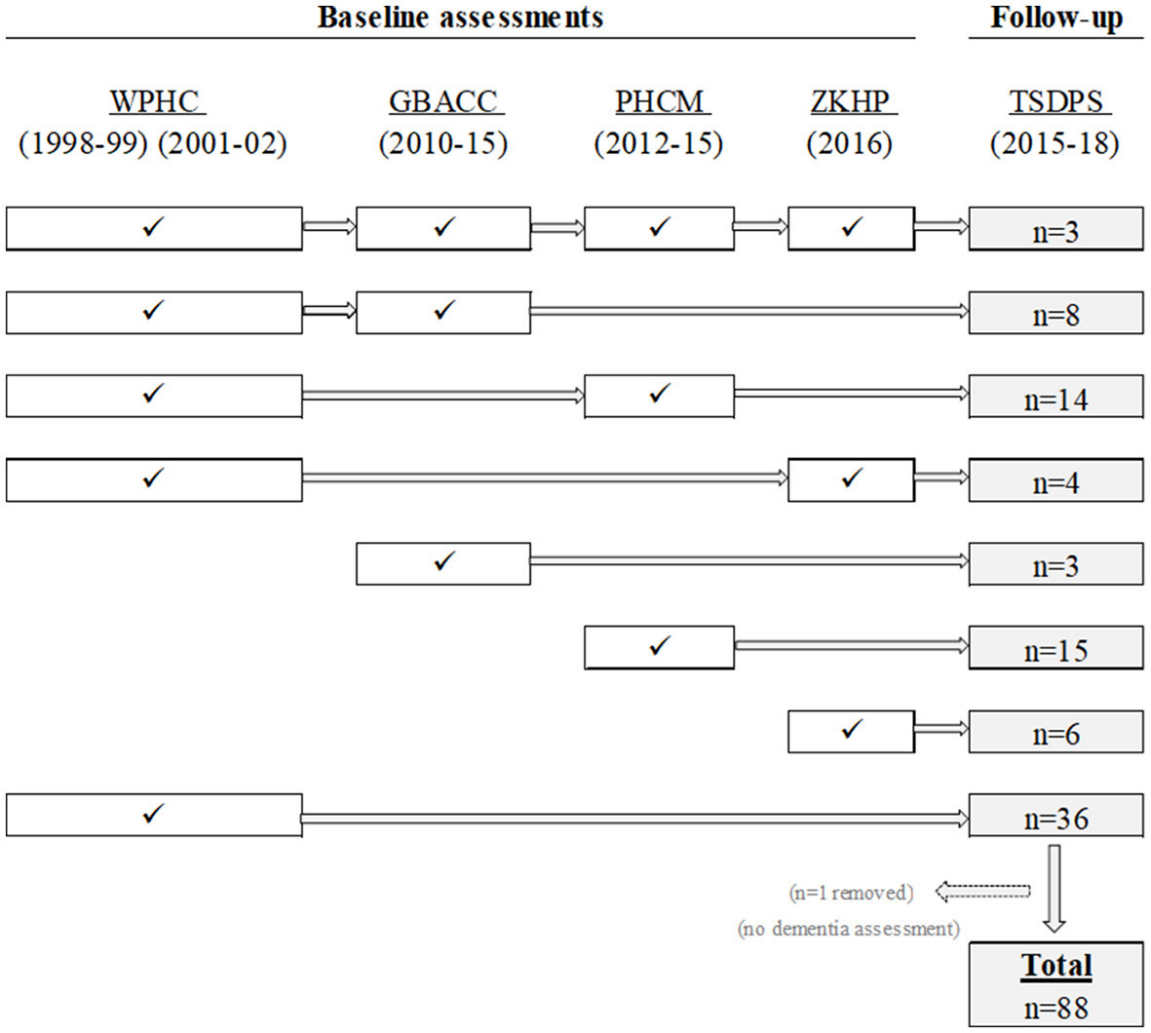
Probabilistic data linkage of people who participated in the Torres Strait Dementia Prevalence Survey (TSDPS) and had at least one baseline measure in the Well Person's Health Check (WPHC), the Getting Better at Chronic Care (GBACC) project, the Primary Health Care Models (PHCM) project, or the Zenadth-Kes Health Partnership (ZKHP) project.

Figure 1 shows that 89 individuals in the TSDPS had at least one health measure in any of the four baseline projects. Forty percent (*n* = 36) of these TSDPS participants only had baseline information from the first and/or second WPHC check. A third (*n* = 29) of TSDPS participants had baseline information from the WPHC and at least one other source (e.g., GBACC, PHCM, or ZKHP). The remaining participants had baseline information from GBACC only (*n* = 3), PHCM (*n* = 15), or the ZKHP (*n* = 6). One participant had missing dementia diagnosis information at follow-up and was excluded from further analyses, resulting in a final sample size of 88 participants.

Supplementary Table 1 shows the number of observations for each health measure across the four baseline studies. The number of observations was determined by how many baseline studies individuals participated in, how many time points they attended in those studies, and how many measures were recorded at these time points. For example, the maximum WPHC time points were two, the GBACC and PHCM had four time points, and the ZKHP had one. While age was recorded for almost all 88 individuals in all baseline studies and time points within these studies (i.e., 285 observations in total), other measures had varying levels of completeness. The most frequently collected measures were T2DM status (279 observations), hypertension, and blood pressure (274 observations, respectively). The least frequently recorded measures were alcohol consumption (153 observations), UACR (194 observations), and VLDL cholesterol (101 observations). Supplementary Table 1 also shows the number of observations when disaggregated by the two time periods of collection prior to the follow-up dementia assessment (i.e., ≤10 years and >10 years).

The median years between baseline observations and the follow-up dementia assessments varied by type of baseline measure (Supplementary Table 3). Most measures were taken 5–12 years before follow-up. HbA1c and diabetes status had the shortest and longest median years (4 and 17, respectively). Measures in the ZKHP were taken the same year as the TSDPS (i.e., a minimum of 0 years before “follow-up”), while some medical records in GBACC included year of first diagnosis of diabetes and hypertension (i.e., maximum of 53 years before “follow-up”).

At follow-up, 33 participants (37.5%) were diagnosed with dementia (*n* = 7) or CIND (*n* = 26) (Table 1 and Supplementary Table 5). A quarter of participants were male (*n* = 24, 27.3%), and a third (*n* = 27, 32.5%) had primary school as the highest level of education. The mean age of participants in the TSDPS (x = 65.9, *SD* = 10.4) was higher compared to the baseline measures (ZKHP, x = 63.8, *SD* = 7.4, PHCM, x = 65.1, *SD* = 10.5, GBACC, x = 57.4, *SD* = 5.5, WPHC, x = 51.7, SD = 10.7) (Supplementary Table 5). The 88 participants in the TSDPS who had participated in at least one baseline study were comparable to other TSDPS participants who did not have baseline data in terms of age (*t* = −1.35, *p* = 0.179) and CIND/dementia status (*chi*^2^ = 0.084, *p* = 0.772), although were more likely to be female (72.7 and 62.2% respectively, *p* = 0.086) (results not tabled).

### 3.2. Risk factors—CIND/dementia status

Table 1 shows that participants with CIND/dementia at follow-up were less likely to have any high school or post-school education (*p* = 0.004) compared to participants without CIND/dementia, although this trend was attenuated when education was dichotomized (*p* = 0.234). In unadjusted analyses, baseline measures of body weight, TG, TC, LDL, and VLDL were lower among participants who developed CIND/dementia, and these associations were observed for the original measures, and with outliers winsorized, or removed. Higher baseline measures of UACR were only associated with CIND/dementia when outliers were winsorized or removed.

### 3.3. Modeling risk factors

Age and time adjusted *meglm* analyses (Table 2) showed that lower body weight (*p* = 0.056) and VLDL (*p* = 0.048) were associated with CIND/dementia. Baseline measures of TG and UACR were only associated with CIND/dementia after winsorizing or removal of outliers (*n* = 9 and *n* = 29, respectively). When only the most two extreme outlying UACR values (i.e., >300) were removed, this measure remained associated with CIND/dementia (*p* = 0.003). Although these associations were statistically significant, their effect sizes (i.e., RRs) were generally modest. Lower cholesterol was significantly associated with CIND/dementia after adjusting for age, although not after adjusting for years between baseline collection and CIND/dementia assessment.

When winsorized baseline measures were examined by time period of collection (i.e., ≤10 years, >10 years), the trends were also unclear (Supplementary Tables 6, 7). Within 10 years of assessment, only UACR remained significant after adjustment for age and years. After 10 years, lower measures of weight, waist circumference and VLDL, and higher UACR, remained trend significant when adjusted for age and years. Sensitivity analyses excluding ZKHP observations (Supplementary Table 8) replicated the main findings in Table 2, although the significance of VLDL reduced to trend level (*p* = 0.070).

## 4. Discussion

This study linked repeated baseline observations with a single follow-up dementia assessment for 88 First Nations residents of the Torres Strait region of Australia. At follow-up, a third of participants (*n* = 33) had CIND (*n* = 26) or dementia (*n* = 7).

We only found a positive association between UACR and later CIND/dementia when outliers were winsorized or removed. However, this association was still observable when only the two most extreme outliers were removed, which provides some evidence of an association between UACR and CIND/dementia. A high UACR reflects cumulative microvascular damage. Our findings concur with published data and suggest that this multisystem microvascular damage, as evidenced in the kidney, is also at play in the cerebral vasculature. Our results were statistically significant; however the small effect size in the final age-adjusted *meglm* analyses makes clinical interpretation less certain. There is substantial evidence that kidney health and brain health are interrelated (Zammit et al., 2016; Deckers et al., 2017) and that a high UACR measures are associated with later increased risk of dementia (Georgakis et al., 2017; Scheppach et al., 2020; Lee S. I. et al., 2022; Lee Y.-B. et al., 2022). Our results add weight to these findings and further reinforce the clinical role of UACR. The UACR is part of the routine Aboriginal and Torres Strait Islander Peoples Health Assessment, known in communities as “Medicare 715” (Australian Government Department of Health Aged Care, 2020). It is a key health assessment tool for this population and is used in primary health services across Australia. When detecting a raised UACR in a patient, primary care health professions should be encouraged to think about dementia prevention. In Australia, the clinical detection and management of UACR in primary care usually triggers a clinical response that is evidence-based (Kidney Health Australia, 2020) with specific guidelines for First Nations Australians (National Aboriginal Community Controlled Health Organisation The Royal Australian College of General Practitioners, 2018). In addition to prevention measures for reducing kidney and cardiovascular disease risk, the focus should also shift to reducing dementia risk, as there is considerable therapeutic overlap.

Lower baseline measures of body weight and VLDL, were associated with later CIND/dementia. Lower TG was only associated with later CIND/dementia after outlying measures were winsorized or excluded. There was no clear trend when these associations were examined in terms of time elapsed between baseline and follow-up (i.e., less/more than 10 years). The results of this study support findings from our previous work. Age-dependant measures of body weight, lipids, and kidney function, collected in primary care, may one day be useful for indicating dementia risk in this First Nations Australian population. Given this data and the spectrum of available published data, interpretation will likely be difficult and specific intervention even less certain.

The results of this study have some similarity to findings documented elsewhere that lower levels of certain lipids, including TC (Peters et al., 2020; Zhu et al., 2022), LDL, and TG (Bernath et al., 2020), were associated with dementia (Lee et al., 2019; Olmastroni et al., 2022). A common interpretation in these studies is that lowered lipids may reflect a decline in preclinical dementia, which occurs in the years before the onset of symptoms and is indicative of other co-occurring processes, such as decreasing nutrition. It is unclear if the associations we observed between lower levels of TG and VLDL reflected this process. For example, we did not have convincing evidence when these lipid measures were assessed closer to CIND/dementia assessment (i.e., within 10 years). Conversely, we found that lower body weight was associated with later CIND/dementia, which is potentially indicative of a general decline (e.g., frailty in pre-clinical dementia) (Peters et al., 2020). So, our finding of lower body weight and lipids may be related to this decline.

The previous studies that found positive associations between early measures of TC, LDL-C (Schilling et al., 2017; Iwagami et al., 2021), and TG (Dimache et al., 2021) with later dementia generally had longer follow-up periods (e.g., ≥7 or ≥10 years). In these studies, high levels of midlife lipids well before a dementia diagnosis were considered as risk factors for developing later dementia rather than reflective of a degenerative process that was already underway. Although we had baseline measures from ≥10 years before dementia assessments, we found no evidence to support higher levels of these lipids as risk factors. Otherwise said, our results do not indicate that high lipid levels had any role in the development of CIND/dementia. Compared to these other studies, an important limitation of our data was that CIND/dementia status was not available when baseline measures were collected. So, despite having baseline measures ≥10 years before a dementia assessment, we were unable to know if a person was cognitively normal and then went on to develop CIND/dementia.

The only lipid associated with later CIND/dementia when measured ≥10 years before the follow-up was VLDL, although the direction of this association was inversed. Compared to other lipids, there is relatively less information about VLDL and dementia risk. Similar results have been found in a study of eight prospective cohorts, where VLDL was inversely associated with dementia risk (Tynkkynen et al., 2018). The authors were unsure if their findings reflected reverse causality, where reduced levels of VLDL indicated subclinical nutritional deficiencies in persons with preclinical CIND/dementia. VLDL is rarely used clinically, as the ultracentrifugation process required to quantify VLDL from plasma is labor intensive (Wilson et al., 1985). Most of our VLDL measures were from one dataset (i.e., the WPHC), and compared to other measures, there were less observations. To obtain more observations, we tested a derived version of VLDL from TG using the formula described in Wilson et al. (1985), and our results matched the TG pattern. Given the limitations of our data, our findings are suggestive, and further research to examine VLDL in larger datasets over more extended periods is required.

The findings of this study may have implications for dementia reduction strategies in First Nations Australians, especially in the Torres Strait and Northern Australia. A substantial proportion of dementia in these populations may be potentially preventable (Ma'u et al., 2021; Thompson et al., 2022a; Sue See et al., 2023). Unlike other populations, there is currently no tailored measure for quantifying future dementia risk and prompting early intervention. The results in the current study may be considered by any future work that seeks to address this gap in clinical services and examine how measures routinely collected in First Nations' primary care could be used to create a dementia risk index. This risk index could then be incorporated into the current national “Medicare 715” routine health assessment.

The results should be interpreted in the context of several important study limitations. Certain variables in the original data (e.g., UACR, TC) had outlying values that potentially affected their associations with CIND/dementia. However, our alternative analyses relied on altered data (i.e., outliers winsorized or removed), so our findings need to be interpreted in the context of this important limitation We also had a small sample size, which was particularly evident when baseline measures were separated into two time periods. The selection of the 10-year cut-off period was made to remain comparable to other research (Iwagami et al., 2021), and it is unclear whether this interval was appropriate for the population of study. There were a small number of dementia cases (*n* = 7) in the follow-up data, so this outcome was combined with CIND (*n* = 26). While CIND and dementia are distinct syndromes, CIND reflects a heightened risk of future dementia and is clinically important in terms of early identification and dementia prevention (Frances et al., 1995). Because secondary data was used, there was no baseline or interval assessments of dementia. As a result, it was not possible to determine whether participants were dementia free during these baseline assessments. As this was a data linkage study, we did not follow a complete cohort, so did not account for the competing risk of death during analyses. Given the long prodromal period of dementia, and the short interval between some of other baseline measures and the follow-up assessment (see Supplementary material), it is likely that some baseline measures were taken during the early preclinical stages of dementia. As a result, the interpretation of findings is limited in terms of whether baseline indicators had an etiological role in the neurodegenerative process. Our analyses were also limited to linear associations, however, the relationship between certain variables and dementia may change depending on stage of life. For example, a higher BMI in midlife is associated with increased dementia risk, while an inverse association has been observed in later life (Brenowitz, 2021). Our findings relate to a unique Australian First Nations population, with its own history and social context distinct from other First Nations peoples internationally and in Australia. Similarly, the sample was overrepresented by females (72.7%). As a result, the generalizability of findings is unclear.

Despite these limitations, the results have some consistency with broader international findings and add weight to the importance of exploring the utility of pathology indicators that are routinely collected in primary care for diagnosing or predicting dementia. Using a data linkage approach with no participant burden demonstrated this method's utility for identifying potentially beneficial information for a population already overburdened by research (Bainbridge et al., 2015; Kris et al., 2021). Future larger-scale work in other populations could leverage a data linkage approach to continue assessing the utility of primary care measures to characterize future dementia risk. This study highlights the lack of knowledge about long-term predictors of dementia specific to First Nations populations, which remains an opportunity for improvement. However, the next step in any related research should ultimately be guided by the communities to address the issues they identify as priorities. For First Nations populations, an emphasis on collecting baseline information on factors known to protect against dementia would assist with changing the narrative of research from a deficits model to a strengths-based approach.

## Data availability statement

Data sharing: The data that support the findings of this study are sensitive. These data are not publicly available due to privacy and ethical restrictions. The data are available on request from the corresponding author. Additional institutional approvals, such as ethics approval, would be required to enable sharing of these data. Requests to access the datasets should be directed to fintan.thompson@jcu.edu.au.

## Ethics statement

The studies involving human participants were reviewed and approved by Far North Queensland Human Research Ethics Committee. Written informed consent for participation was not required for this study in accordance with the national legislation and the institutional requirements.

## Author contributions

FT conceived the research, obtained approvals to combine study data from the five projects, assisted with data collection for several projects, linked the datasets, analyzed the data, interpreted the results, and prepared the manuscript. SR, RQ, MM, SC, ST, LH, BSa, BSc, GM, and PM assisted with data collection and interpretation of the data and contributed to the manuscript. ST and BSa provided cultural advice and assisted with stakeholder consultations before and during data collection. AE provided statistical advice. ES and RM provided medical advice, oversaw the projects, and reviewed the manuscript. All authors contributed to the article and approved the submitted version.
