# Supplementary material for: Primary care biomarkers and dementia in people of the Torres Strait, Australia: extended data analysis
Source: Front Dement. 2023 Jul 31;2:1218709. doi: 10.3389/frdem.2023.1218709 (PMC11285673; doi:10.3389/frdem.2023.1218709)
Supplement: Supplementary file 1 [file Data_Sheet_1.DOCX]

**Supplementary File 1**

**Figure 1 – Distribution of dates of triglyceride observations for 88 of people who participated in the Dementia Prevalence Survey (DPS) and had at least one baseline triglyceride measure in the Well Person’s Health Check (WPHC), the Getting Better at Chronic Care (GBACC) project, the Primary Health Care Models (PHCM) project, or the Zenadth-Kes Health Partnership (ZKHP) project**


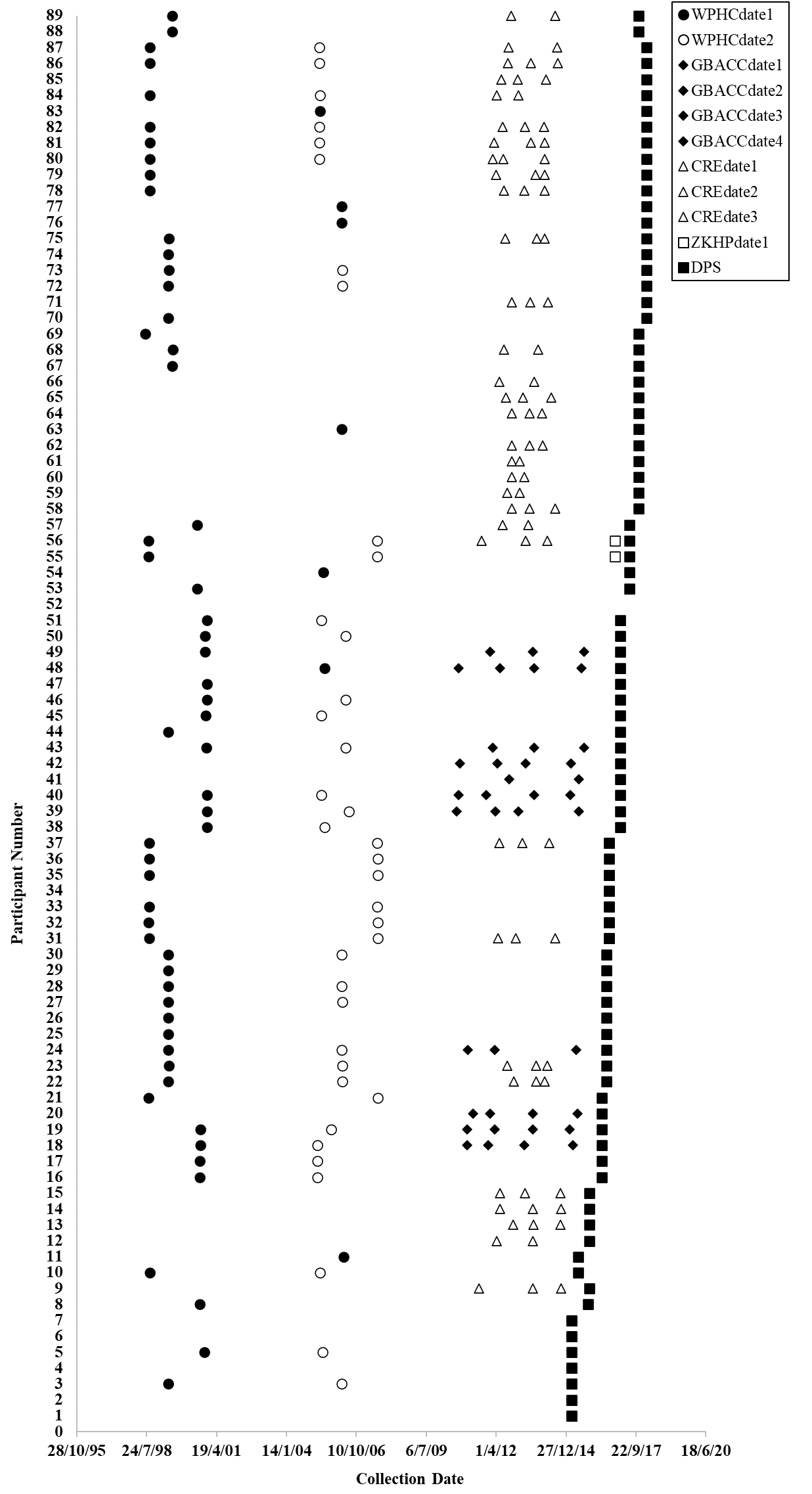


**Figure 2 – Anonymized example structure of data used for analyses** **for 88 of people who participated in the Dementia Prevalence Survey and had at least one baseline measure**
