# Supplementary figures and images for: Primary care biomarkers and dementia in people of the Torres Strait, Australia: extended data analysis
Source: Front Dement. 2023 Jul 31;2:1218709. doi: 10.3389/frdem.2023.1218709 (PMC11285673; doi:10.3389/frdem.2023.1218709)

Figure 1

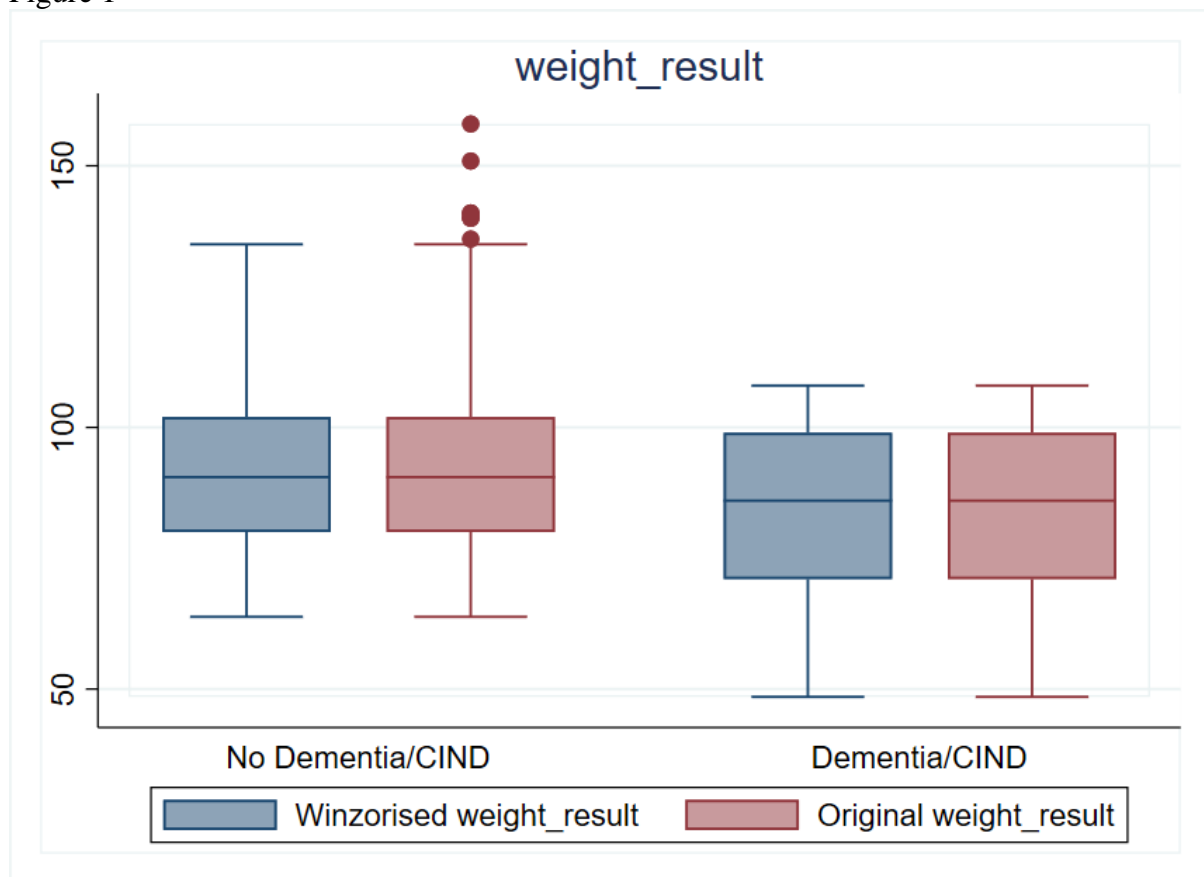

Figure 2

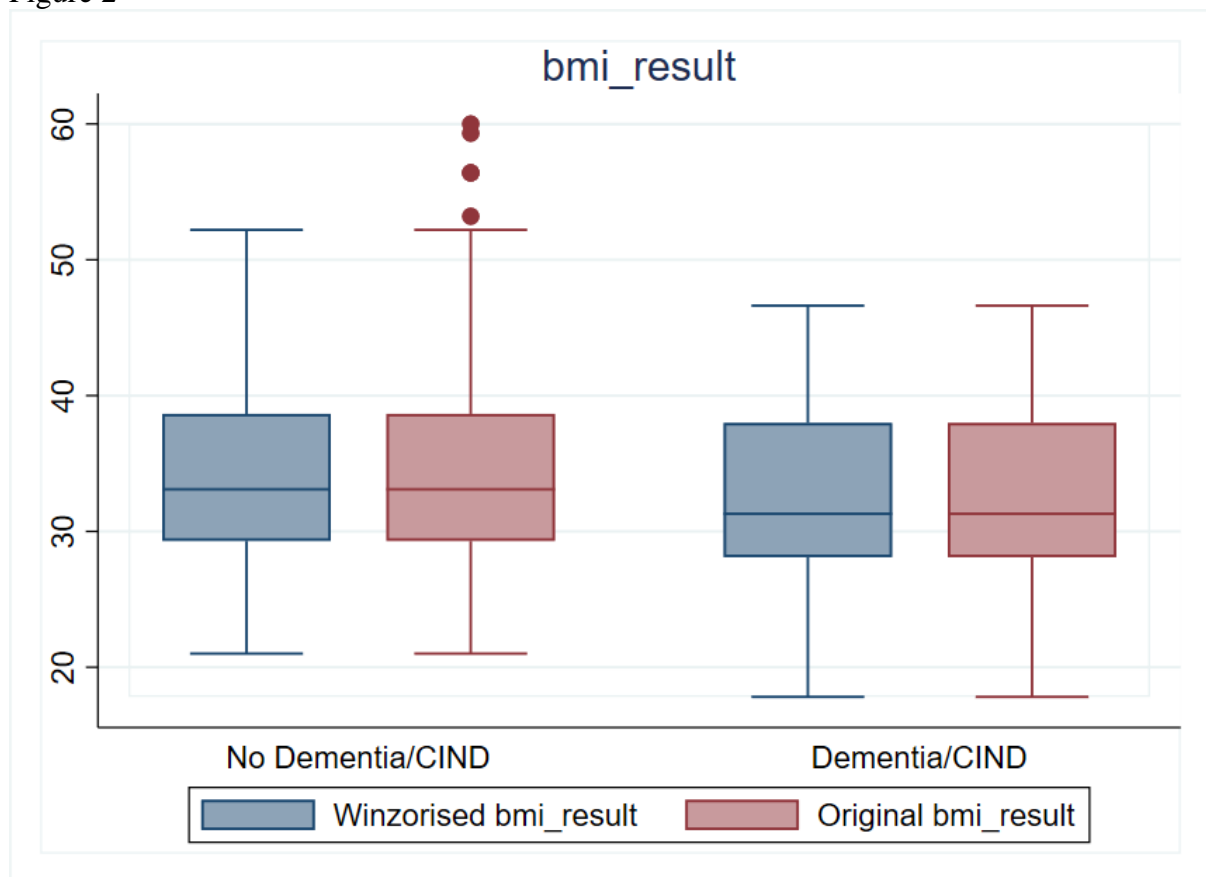

Figure 3

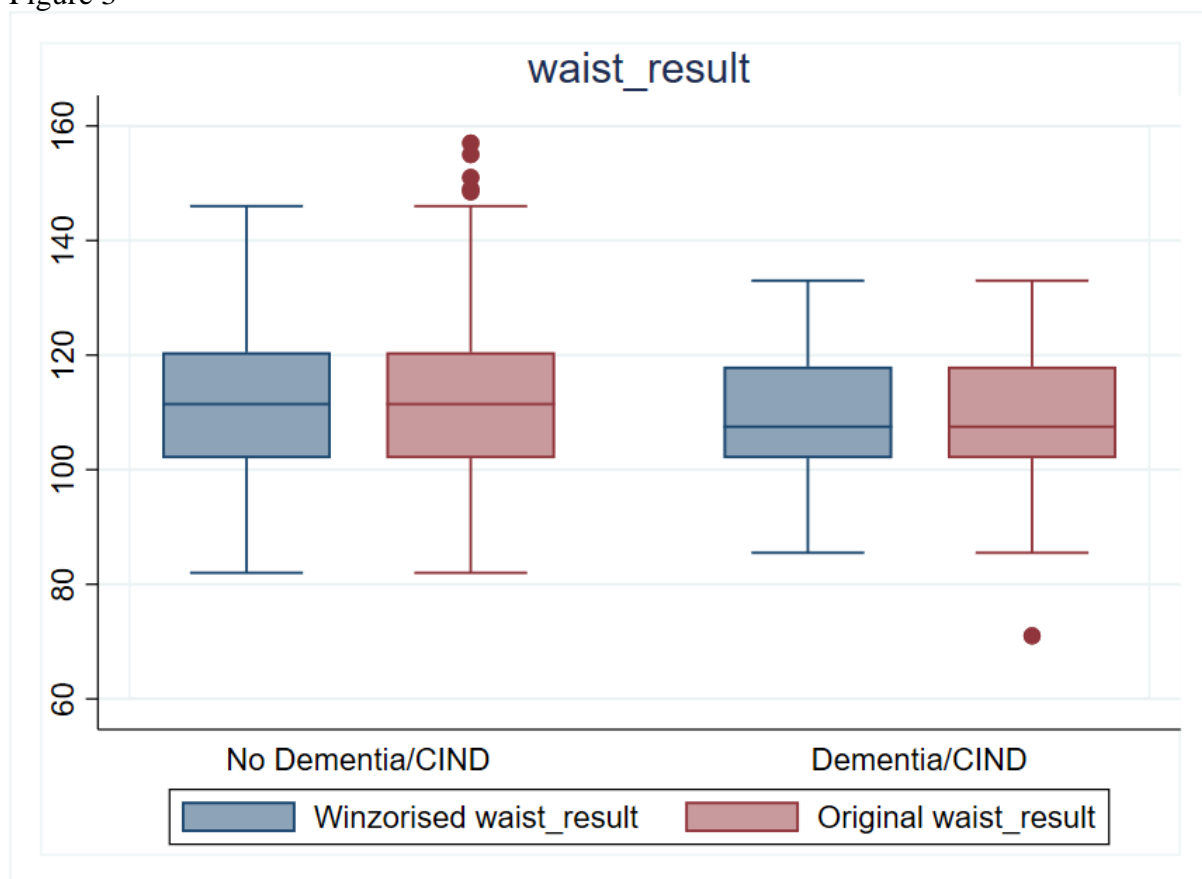

Figure 4

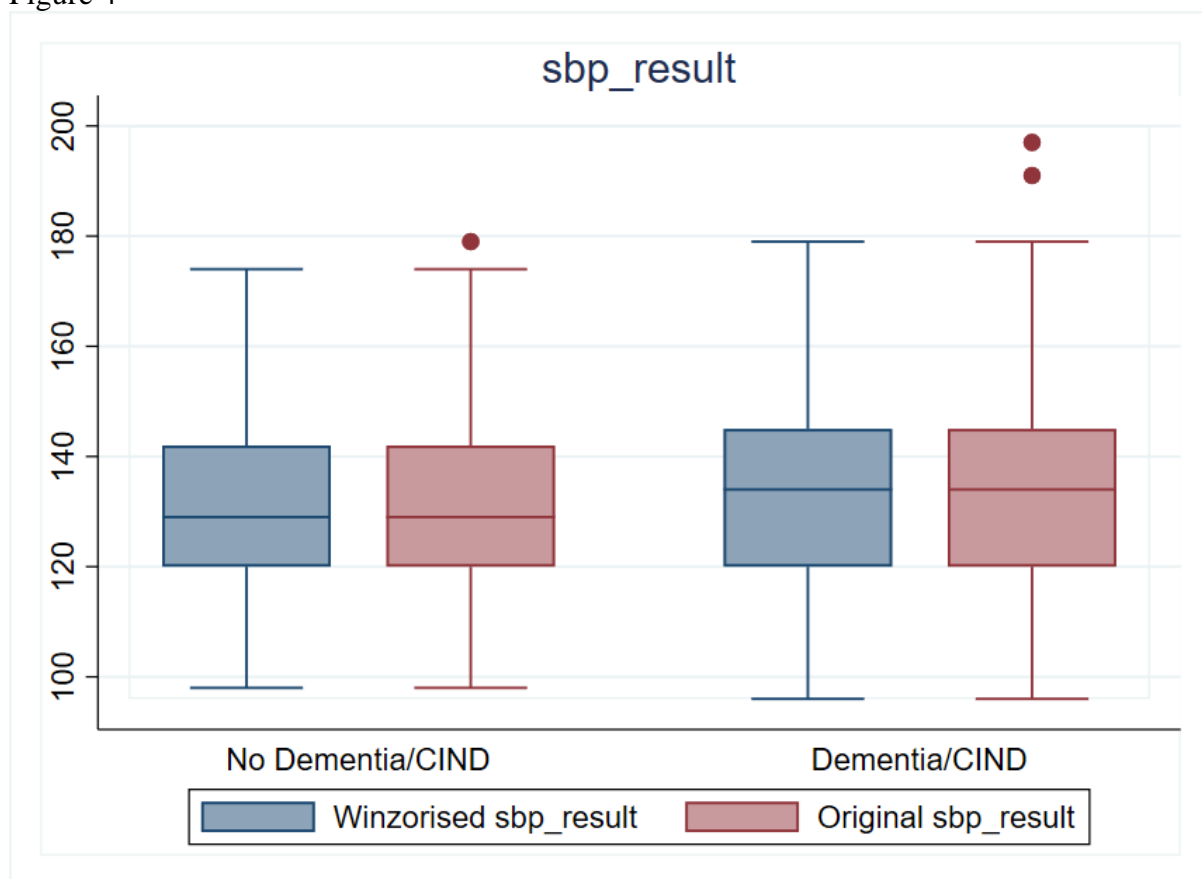

Figure 5

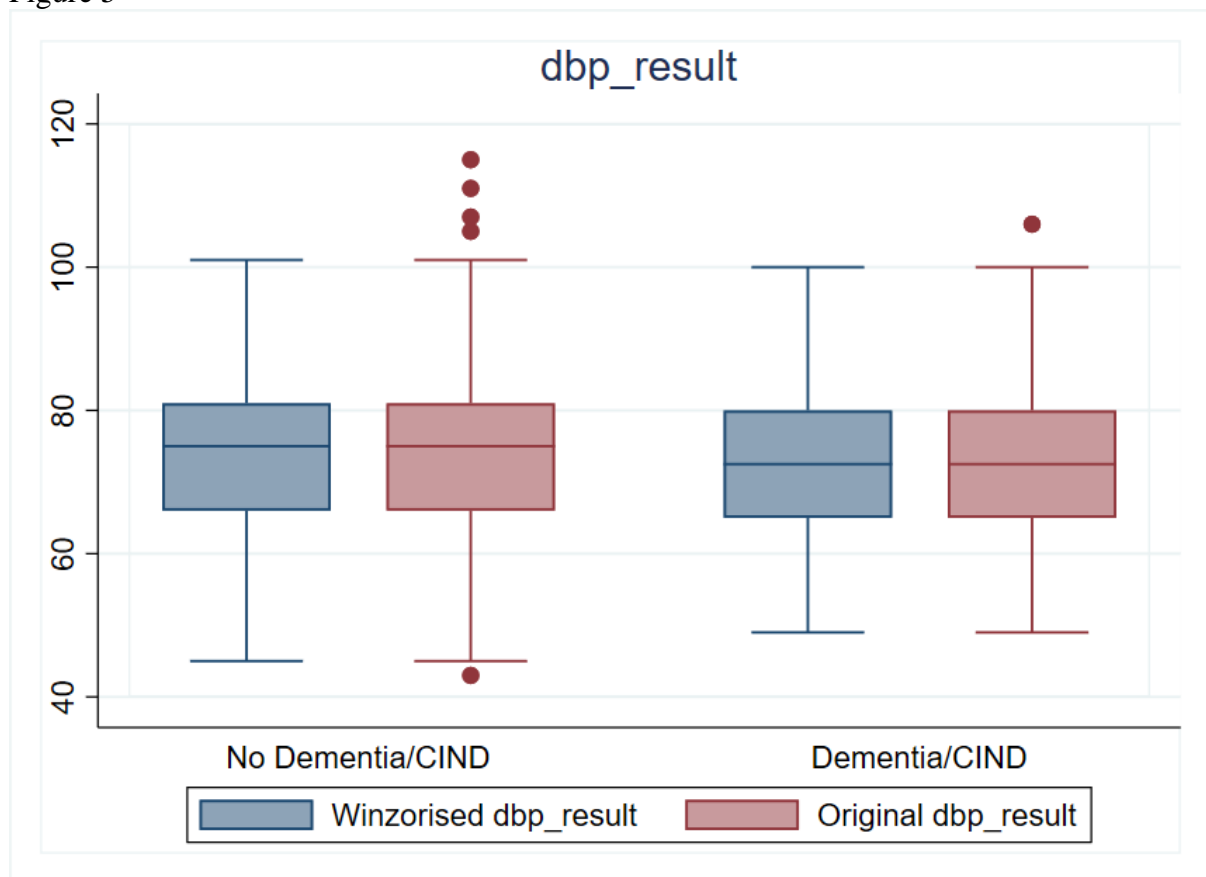

Figure 6

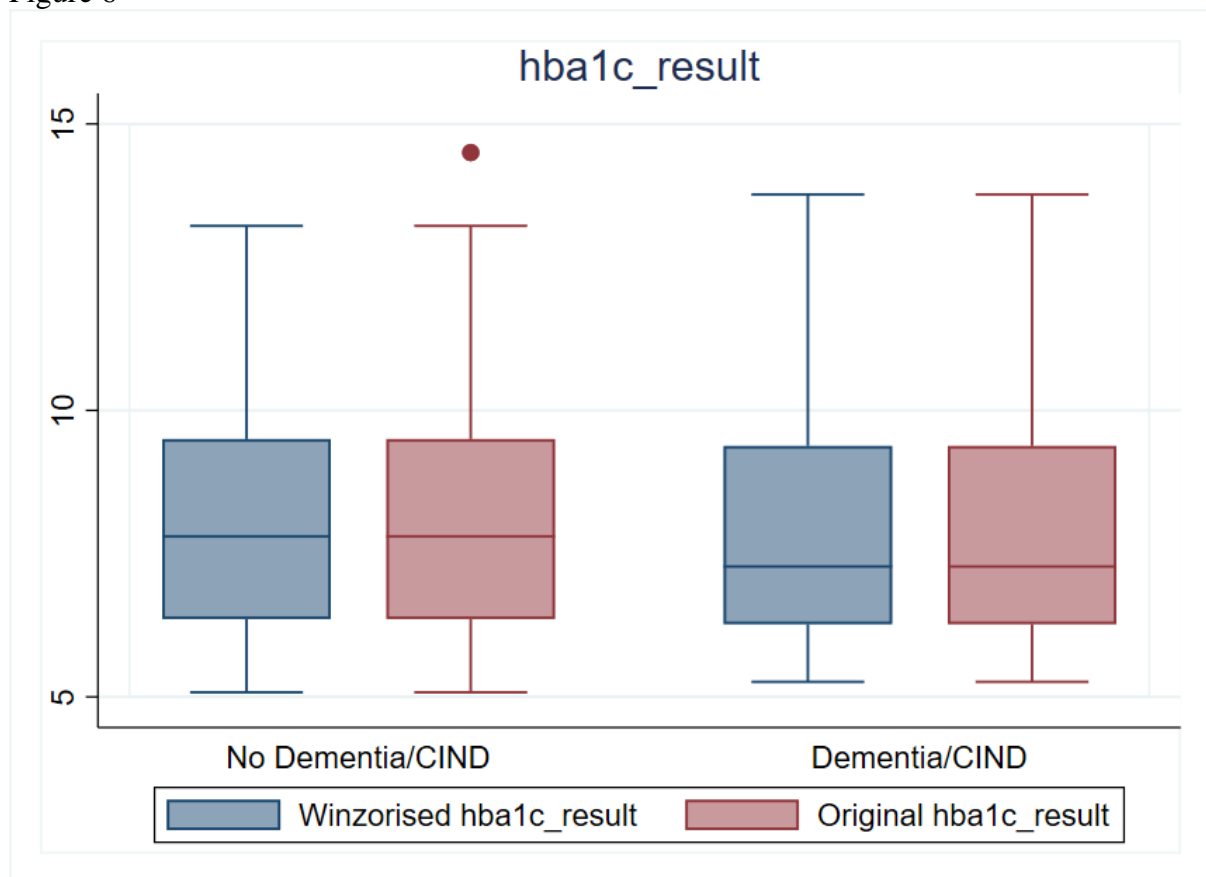

Figure 7

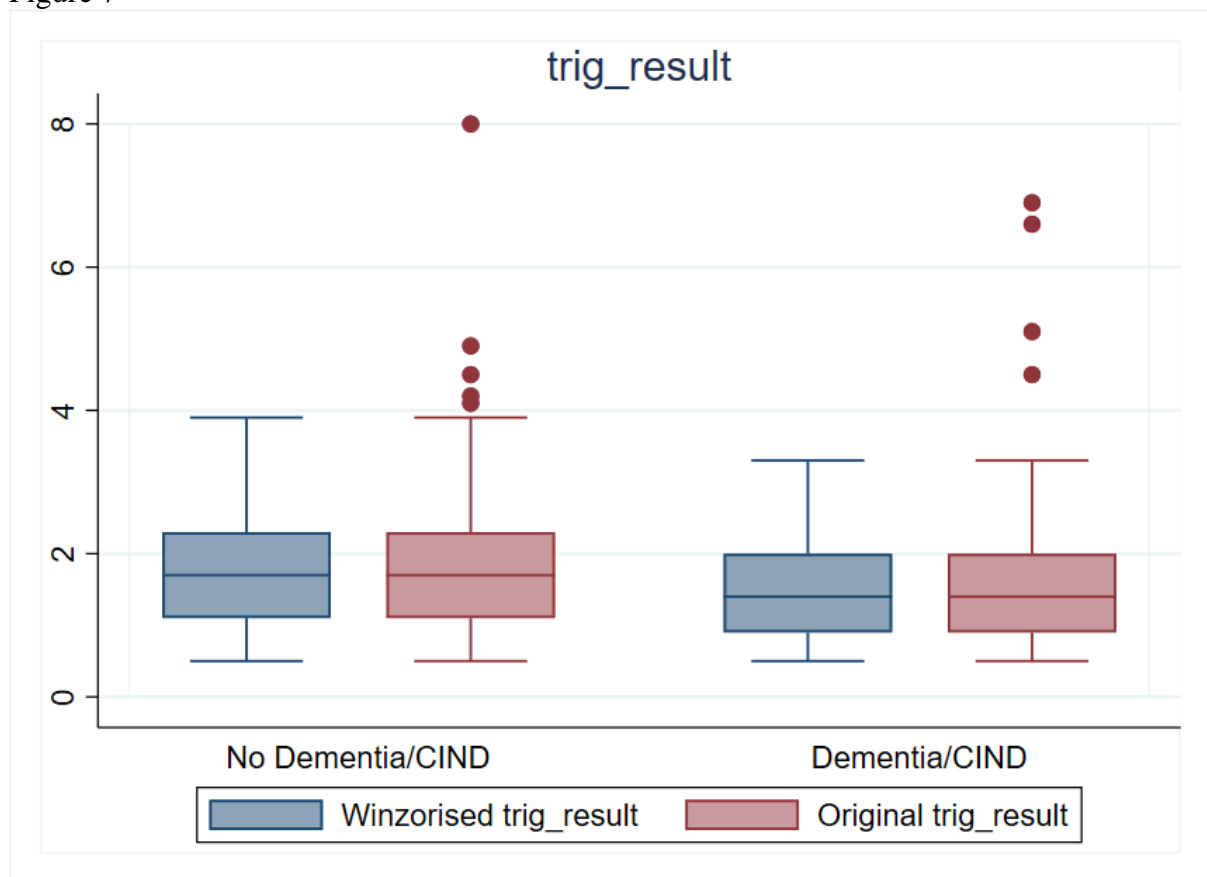

Figure 8

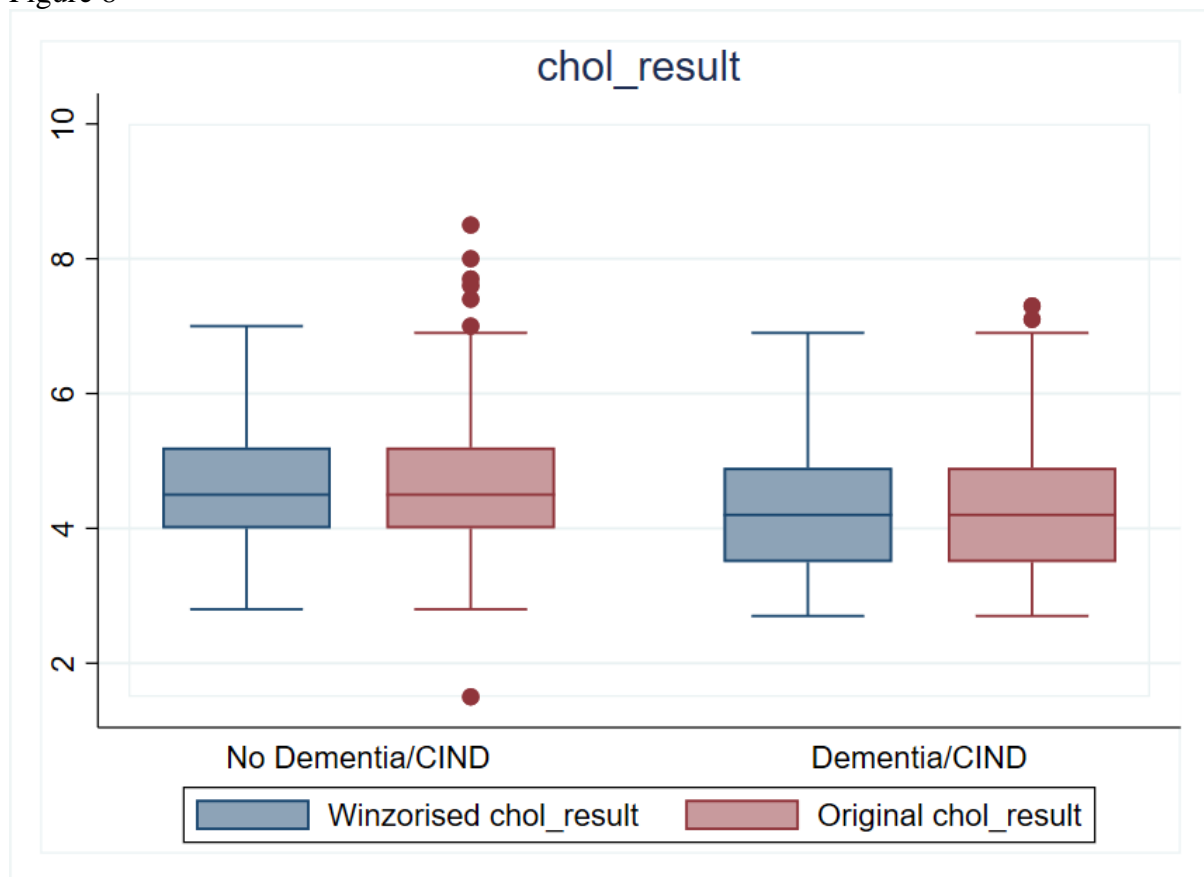

Figure 9

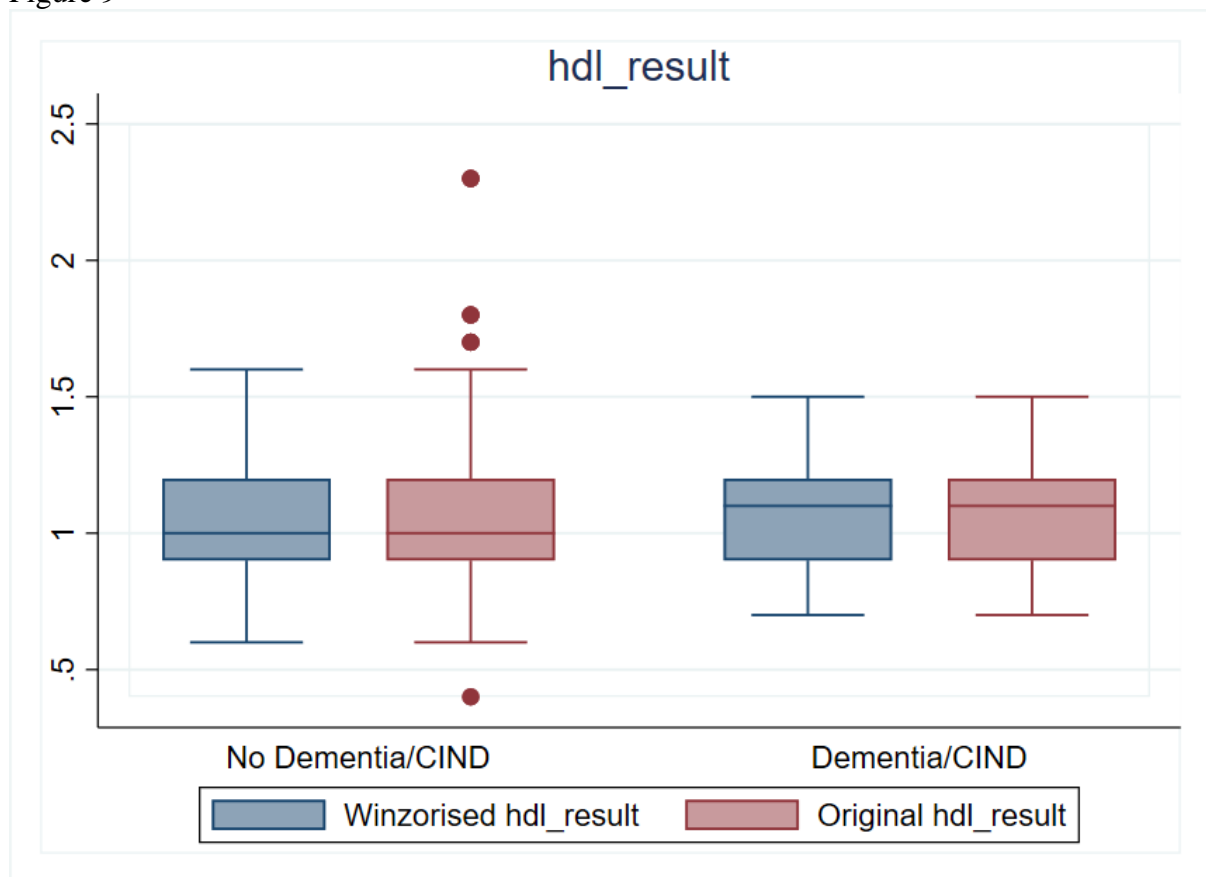

Figure 10

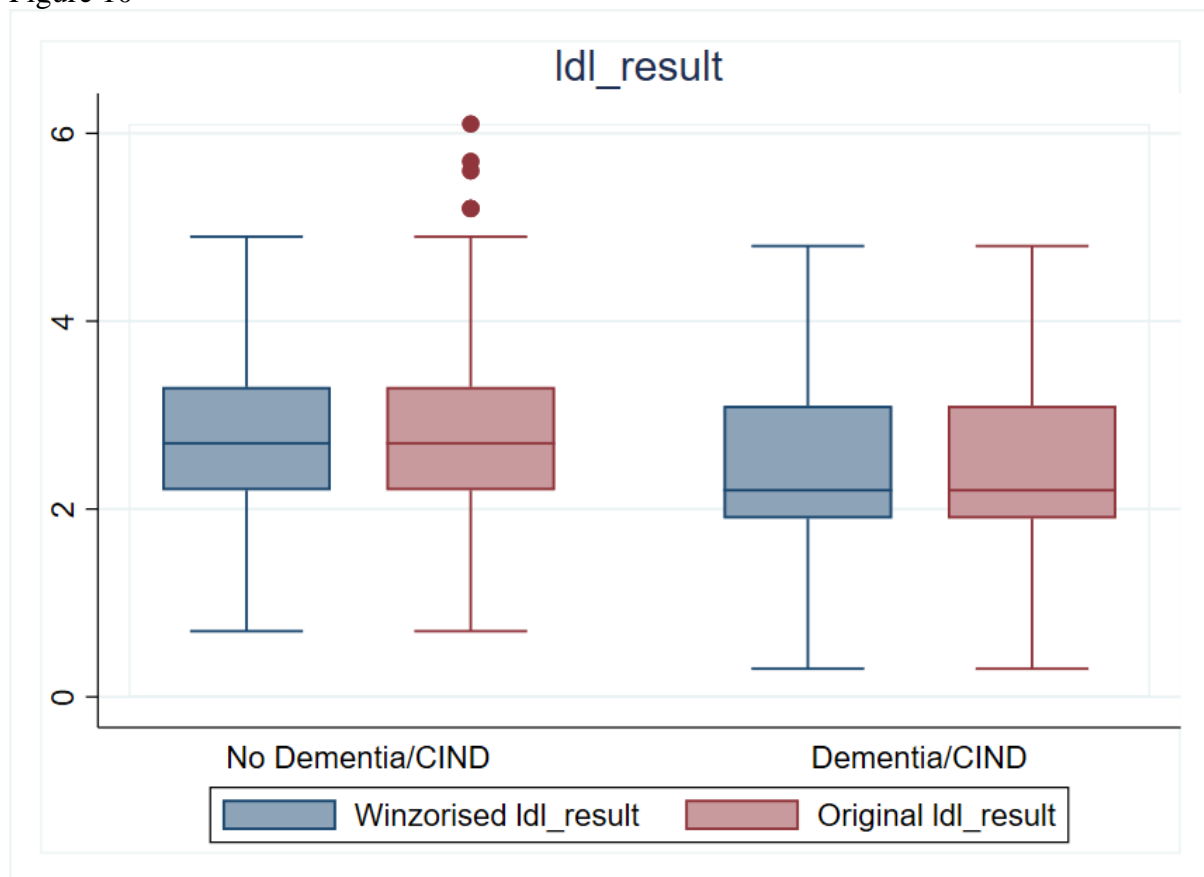

Figure 11

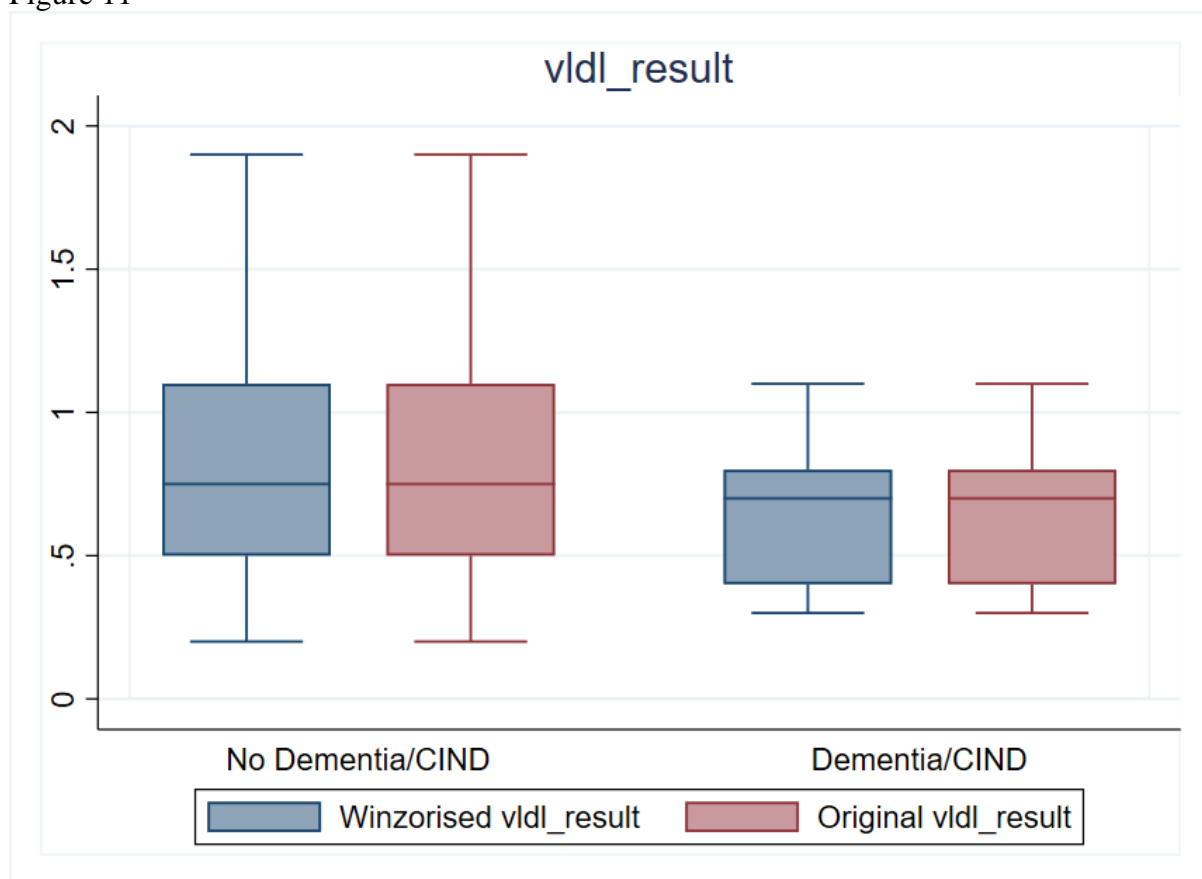

Figure 12

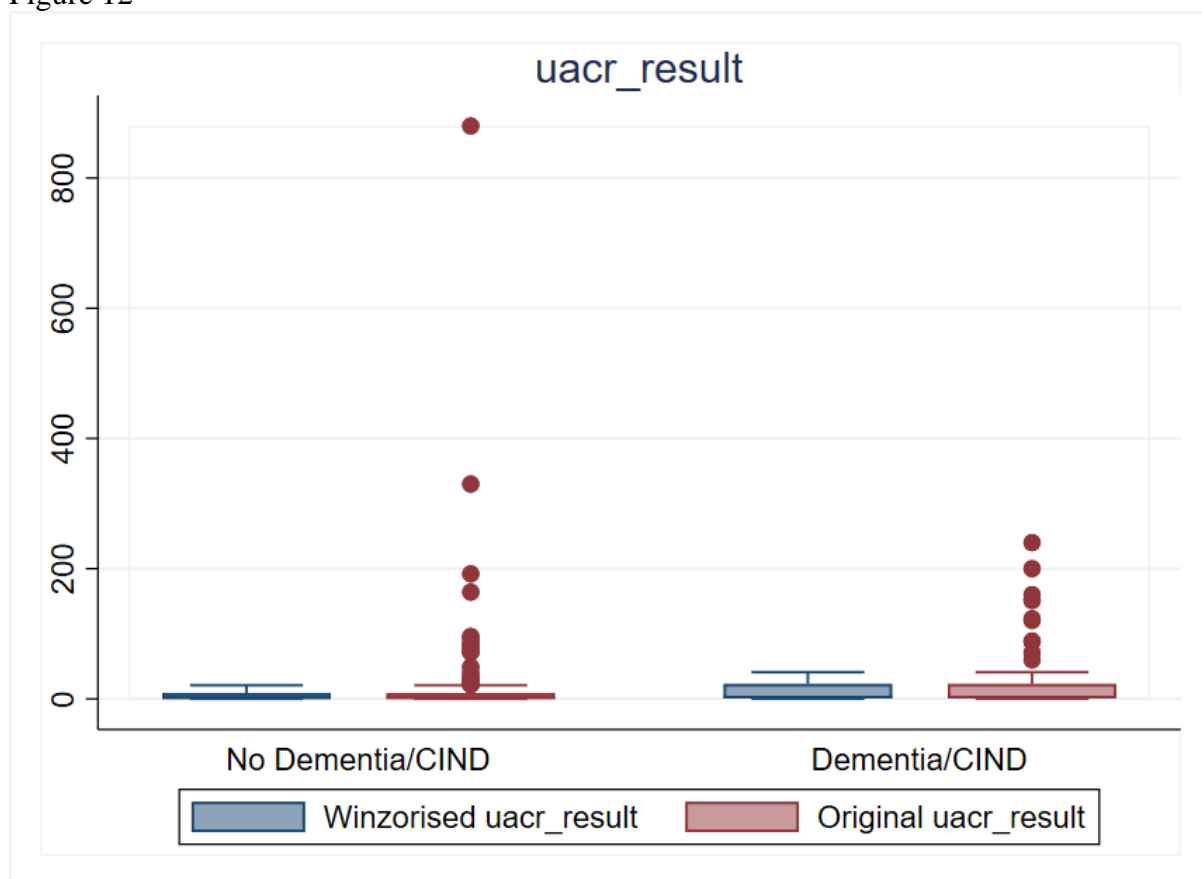

Figure 13

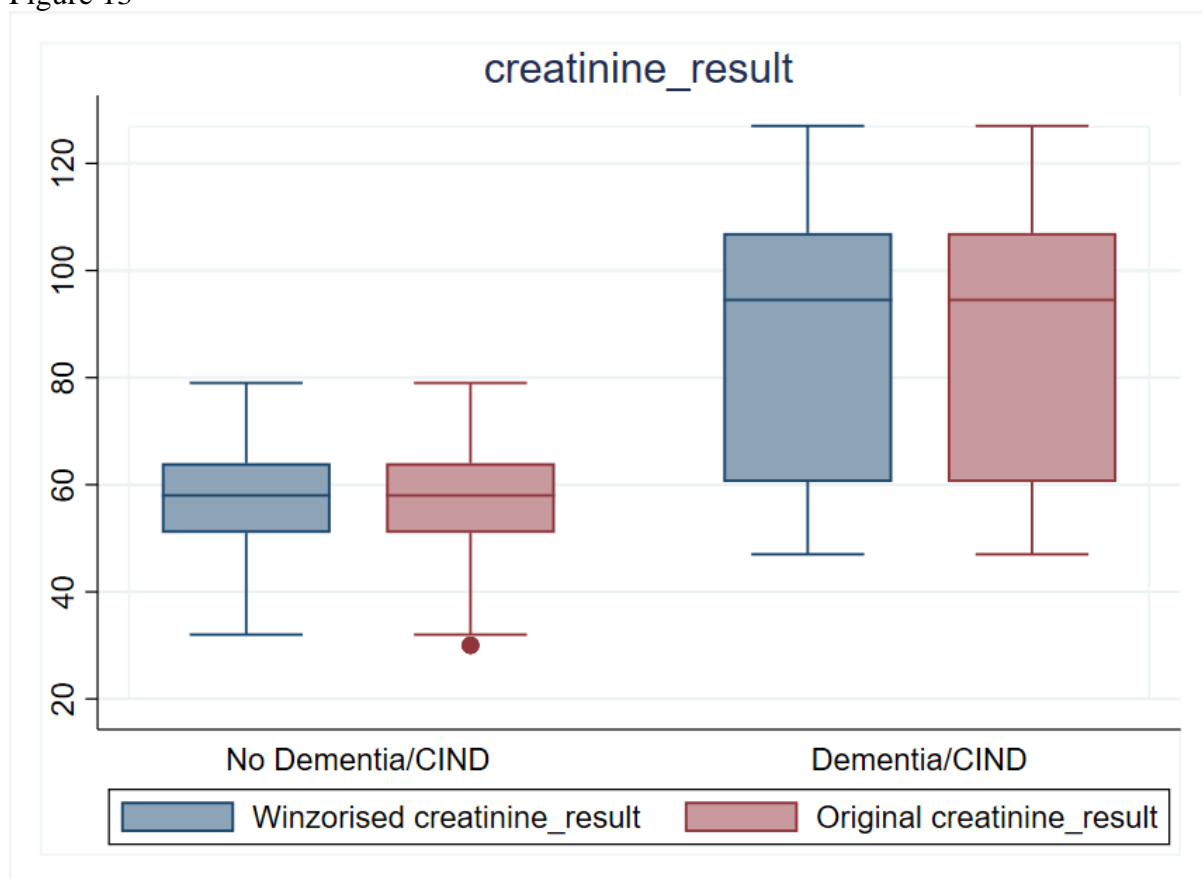

Supplement: Supplementary file 2 [file Data_Sheet_2.PDF]
