# Supplementary material for: Primary care biomarkers and dementia in people of the Torres Strait, Australia: extended data analysis
Source: Front Dement. 2023 Jul 31;2:1218709. doi: 10.3389/frdem.2023.1218709 (PMC11285673; doi:10.3389/frdem.2023.1218709)
Supplement: Supplementary file 3 [file Data_Sheet_3.DOCX]

**Supplementary File 3 – Sensitivity Analyses**

**Generalized Estimating Equations (GEE)**

**Table 1 – Generalized Estimating Equations (GEE) with relative risk (RR) associations between baseline measures and a diagnosis of Cognitive Impairment No Dementia (CIND) or dementia at follow-up for 88 people who participated in at least one baseline study, unadjusted and adjusted for age**

| **Variables** |  | **CIND or Dementia** |  | **Unadjusted** | | |  | **Adjusted - Age** | | |
| --- | --- | --- | --- | --- | --- | --- | --- | --- | --- | --- |
|  | **N** | **No / Yes** |  | **RR** | **(95%CI)** | **p** |  | **RR** | **(95%CI)** | **p** |
| **Categorical** |  |  |  |  |  |  |  |  |  |  |
| Smoking (Ref: No) | 244 | 39 / 10 |  | 0.98 | (0.95-1.02) | 0.298 |  | 0.98 | (0.85-1.12) | 0.739 |
| Alcohol (Ref: No) |  | 48 / 14 |  |  |  |  |  |  |  |  |
| Diabetes (Ref: No) | 279 | 135 / 84 |  | 1.01 | (0.99-1.04) | 0.357 |  | 1.03 | (0.93-1.13) | 0.600 |
| Hypertension (Ref: No) | 274 | 126 / 88 |  | 1.01 | (1.00-1.02) | 0.050 |  | 1.01 | (0.97-1.05) | 0.634 |
| UACR (≥14) (Ref: No) |  | 22 / 25 |  |  |  |  |  | 1.02 | (0.98-1.07) | 0.248 |
| Triglycerides (≤0.8 mmol/L) (Ref: No) | 237 | 17 / 19 |  | 1.01 | (1.00-1.03) | 0.076 |  | 1.08 | (1.00-1.17) | 0.049 |
| Cholesterol (≤3.5 mmol/L) (Ref: No) | 263 | 19 / 32 |  | 1.01 | (1.00-1.02) | 0.021 |  | 1.05 | (1.01-1.10) | 0.019 |
| LDL (≤1.7 mmol/L) (Ref: No) | 252 | 20 / 19 |  | 1.00 | (1.00-1.01) | 0.193 |  | 1.03 | (0.99-1.08) | 0.119 |
| **Continuous** |  |  |  |  |  |  |  |  |  |  |
| Weight (kg) | 266 | 167 / 99 |  | 1.00 | (1.00-1.00) | 0.004 |  | 0.99 | (0.99-1.00) | 0.045 |
| BMI | 241 | 152 / 89 |  | 1.00 | (0.99-1.00) | 0.206 |  | 1.00 | (0.98-1.01) | 0.648 |
| Waist (cm) | 204 | 128 / 76 |  | 1.00 | (1.00-1.00) | 0.436 |  | 1.00 | (0.99-1.00) | 0.437 |
| Systolic BP (mmHg) | 274 | 176 / 98 |  | 1.00 | (1.00-1.00) | 0.475 |  | 1.00 | (1.00-1.00) | 0.940 |
| Diastolic BP (mmHg) | 274 | 176 / 98 |  | 1.00 | (1.00-1.00) | 0.361 |  | 1.00 | (1.00-1.00) | 0.187 |
| HbA1c (% NGSP) | 216 | 137 / 79 |  | 1.00 | (1.00-1.00) | 0.819 |  | 1.00 | (1.00-1.00) | 0.131 |
| UACR (ratio) |  | 118 / 76 |  |  |  |  |  | 1.00 | (1.00-1.00) | 0.137 |
| Triglycerides (mmol/L) | 237 | 150 / 87 |  | 0.99 | (0.99-1.00) | 0.024 |  | 0.96 | (0.92-0.99) | 0.020 |
| Cholesterol (mmol/L) | 263 | 166 / 97 |  | 1.00 | (0.99-1.00) | 0.057 |  | 0.98 | (0.95-1.00) | 0.043 |
| HDL (mmol/L) | 263 | 166 / 97 |  | 1.00 | (0.98-1.03) | 0.784 |  | 0.99 | (0.87-1.13) | 0.914 |
| LDL (mmol/L) | 252 | 162 / 90 |  | 1.00 | (0.99-1.00) | 0.058 |  | 0.98 | (0.95-1.00) | 0.075 |
| VLDL (mmol/L) | 101 | 76 / 25 |  | 0.50 | (0.31-0.81) | 0.005 |  | 0.41 | (0.21-0.80) | 0.009 |

**Table 2 – Generalized Estimating Equations with relative risk (RR) associations between selected baseline measures and a diagnosis of Cognitive Impairment No Dementia (CIND) or dementia at follow-up for 88 people who participated in at least one baseline study, unadjusted and adjusted for age**

| **Variables** |  | **CIND or Dementia** |  | **Unadjusted** | | |  | **Adjusted - Age** | | |
| --- | --- | --- | --- | --- | --- | --- | --- | --- | --- | --- |
|  | **N** | **No / Yes** |  | **RR** | **(95%CI)** | **p** |  | **RR** | **(95%CI)** | **p** |
|  |  |  |  |  |  |  |  |  |  |  |
| Whole time period |  |  |  |  |  |  |  |  |  |  |
| Triglycerides (≤0.8 mmol/L) (Ref: No) | 237 | 17 / 19 |  | 1.01 | (1.00-1.03) | 0.076 |  | 1.08 | (1.00-1.17) | 0.049 |
| Cholesterol (≤3.5 mmol/L) (Ref: No) | 263 | 19 / 32 |  | 1.01 | (1.00-1.02) | 0.021 |  | 1.05 | (1.01-1.10) | 0.019 |
| Weight (kg) | 266 | 167 / 99 |  | 1.00 | (1.00-1.00) | 0.004 |  | 0.99 | (0.99-1.00) | 0.045 |
| Triglycerides (mmol/L) | 237 | 150 / 87 |  | 0.99 | (0.99-1.00) | 0.024 |  | 0.96 | (0.92-0.99) | 0.020 |
| Cholesterol (mmol/L) | 263 | 166 / 97 |  | 1.00 | (0.99-1.00) | 0.057 |  | 0.98 | (0.95-1.00) | 0.043 |
| LDL (mmol/L) | 252 | 162 / 90 |  | 1.00 | (0.99-1.00) | 0.058 |  | 0.98 | (0.95-1.00) | 0.075 |
| VLDL (mmol/L) | 101 | 76 / 25 |  | 0.50 | (0.31-0.81) | 0.005 |  | 0.41 | (0.21-0.80) | 0.009 |
|  |  |  |  |  |  |  |  |  |  |  |
| ≥10 years |  |  |  |  |  |  |  |  |  |  |
| Weight (kg) | 90 | 64 / 26 |  | 0.98 | (0.96-0.99) | 0.002 |  | 0.98 | (0.96-1.00) | 0.045 |
| UACR (ratio) | 80 | 56 / 24 |  | 1.02 | (1.00-1.04) | 0.026 |  | 1.02 | (1.00-1.03) | 0.045 |
| VLDL (mmol/L) | 80 | 59 / 21 |  | 0.43 | (0.25-0.76) | 0.003 |  | 0.35 | (0.16-0.76) | 0.008 |

**Notes:** Within 10 years≤0.0-10.0 years, After 10 years>10.0 years, Weight=body weight (kilograms, kg), HbA1c=hemoglobin A1c (%), UACR=Urinary albumin creatinine ratio, VLDL=very low-density lipoprotein.
