# Supplementary material for: Primary care biomarkers and dementia in people of the Torres Strait, Australia: extended data analysis
Source: Front Dement. 2023 Jul 31;2:1218709. doi: 10.3389/frdem.2023.1218709 (PMC11285673; doi:10.3389/frdem.2023.1218709)
Supplement: Supplementary file 4 [file Table_1.docx]

**Supplementary Box 1 – List of tables and figures, including titles, purpose and main findings**

| **Manuscript Tables and Figures** | | |
| --- | --- | --- |
|  | **Title** | **Plain language description of purpose** |
| **Figure 1** | Probabilistic data linkage of people who participated in the Torres Strait Dementia Prevalence Survey (TSDPS) and had at least one baseline measure in the Well Person’s Health Check (WPHC), the Getting Better at Chronic Care (GBACC) project, the Primary Health Care Models (PHCM) project, or the Zenadth-Kes Health Partnership (ZKHP) project | This is a graphical display of the data linkage and the number of individuals across different baseline studies. |
| **Table 2** | Distribution of baseline variables by CIND/dementia status at follow-up for 88 individuals who participated in the Torres Strait Dementia Prevalence Survey (TSDPS) with at least one baseline assessment | This table shows the distribution of individuals and observations by dementia status at follow-up. Categorical variables are summarised as percentages and continuous variables as measures of central tendency. Statistical differences between baseline measures by dementia status are tested. |
| **Table 3** | Mixed Effects Generalized Linear Modelling (meglm) with relative risk (RR) associations between selected baseline measures and a diagnosis of Cognitive Impairment No Dementia (CIND) or dementia at follow-up for 88 people who participated in at least one baseline study, unadjusted and adjusted for age | This table shows the main analyses of the paper. The differences in baseline observations, by follow-up dementia status is tested, after accounting for repeat observations for the same individual. Results are displayed for the entire time period of baseline data, and by two periods (i.e., ≤10 years, and >10 years). Only selected results are presented.  The count data underlying these results are provided in Table2 for the whole time period. Similarly, Supplementary Table 7 contains the count data underlying these results for the two time periods separately. Supplementary Table 9 contains all results from these analyses, for the whole time period. |
| **Supplementary Tables and Figures** | | |
| **Figure 1** | Availability of variables across the Well Person’s Health Check (WPHC), the Getting Better at Chronic Care (GBACC) project, the Primary Health Care Models (PHCM) project, the Zenadth-Kes Health Partnership (ZKHP) project and the Torres Strait Dementia Prevalence Survey (TSDPS) | This is a graphical display of available variables, by different baseline studies. |
| **Table 1** | Completeness of variables across the Well Person’s Health Check (WPHC), the Getting Better at Chronic Care (GBACC) project, the Primary Health Care Models (PHCM) project, the Zenadth-Kes Health Partnership (ZKHP) project and the Torres Strait Dementia Prevalence Survey (TSDPS) | This is a numerical display of the observations per variable, by different baseline studies. |
| **Table 2** | Number of winsorized observations across the Well Person’s Health Check (WPHC), the Getting Better at Chronic Care (GBACC) project, the Primary Health Care Models (PHCM) project and the Zenadth-Kes Health Partnership (ZKHP) | This is a numerical display of the effect of winsorizing variables to remove outliers. |
| **Table 3** | Years between baseline measures from linked datasets and follow-up of individuals who participated in the Torres Strait Dementia Prevalence Survey (TSDPS) | This is a numerical display of the time between different baseline measures and follow-up dementia assessment. This table also shows the ‘spread’ in time, through measures of central tendency. |
| **Table 4** | Completeness of variables by time of measurement across the Well Person’s Health Check (WPHC), the Getting Better at Chronic Care (GBACC) project, the Primary Health Care Models (PHCM) project, the Zenadth-Kes Health Partnership (ZKHP) project and the Torres Strait Dementia Prevalence Survey (TSDPS) | This is a numerical display of the observations per variable, by different baseline studies, by time period (i.e., ≤10 years and >10 years). This table shows that most of the baseline observations were within 10 years of dementia assessments. |
| **Table 5** | Distribution of baseline variables from linked datasets of 88 individuals who participated in the Torres Strait Dementia Prevalence Survey (TSDPS) with at least one baseline assessment | This table has summary measures of the different baseline variables, to show how they varied across categories (e.g., yes/no) or their distribution (e.g., mean, median). |
|  |  |  |
| **Table 6** | Distribution of baseline variables by timing of collection and CIND/dementia status at follow-up for 88 individuals who participated in the Torres Strait Dementia Prevalence Survey (TSDPS) and at least one baseline assessment | This table displays the distribution of baseline variables by follow-up dementia status, by time period (i.e., ≤10 years and >10 years). Only results for selected variables are presented. This table provides underlying numbers for the results in Manuscript Table 3. |
|  |  |  |
| **Table 7** | Mixed Effects Generalized Linear Modelling (meglm) with relative risk (RR) associations between baseline measures and a diagnosis of Cognitive Impairment No Dementia (CIND) or dementia at follow-up for 88 people who participated in at least one baseline study, unadjusted and adjusted for age | This table shows differences in baseline observations, by follow-up dementia status. The results account for repeat observations for the same individual. Selected results are displayed for the whole time period and for >10 years. |
| **Table 8** | Main analyses excluding ZKHP participants (n=13), Mixed Effects Generalized Linear Modelling (meglm) with relative risk (RR) associations between selected baseline measures and a diagnosis of Cognitive Impairment No Dementia (CIND) or dementia at follow-up for 88 people who participated in at least one baseline study, adjusted for age and years between measure and follow-up | This table reports sensitivity analyses for the main results, by excluding observations from people who participated in the ZKHP. |
|  |  |  |

**Supplementary Figure 1 – Availability of variables across the Well Person’s Health Check (WPHC), the Getting Better at Chronic Care (GBACC) project, the Primary Health Care Models (PHCM) project, the Zenadth-Kes Health Partnership (ZKHP) project and the Torres Strait Dementia Prevalence Survey (TSDPS)**

INSERT SUPPLEMENTARY FIGURE 1 HERE

**Notes:** CIND=Cognitive Impairment No Dementia, Weight=body weight (kilograms, kg), BMI=Body Mass Index [weight(kg)/height2], Waist=waist circumference (centimetres, cm), Smoking= self-reported “currently smoked tobacco,” Alcohol=self-reported any alcohol consumption (Yes/No), Systolic BP=systolic blood pressure, Diastolic BP=diastolic blood pressure, HbA1c=haemoglobin A1c (%), UACR=Urinary albumin creatinine ratio, HDL=high-density lipoprotein, LDL=low-density lipoprotein, VLDL=very low-density lipoprotein.

**Supplementary Table 1 – Completeness of variables across the Well Person’s Health Check (WPHC), the Getting Better at Chronic Care (GBACC) project, the Primary Health Care Models (PHCM) project, the Zenadth-Kes Health Partnership (ZKHP) project and the Torres Strait Dementia Prevalence Survey (TSDPS)**

|  |  | **Baseline assessments** | | | | |  | **Follow-up** |  | **Years before follow-up** | |
| --- | --- | --- | --- | --- | --- | --- | --- | --- | --- | --- | --- |
| **Variables** |  | WPHC  (1998-99) (2001-02) | GBACC  (2010-15) | PHCM  (2012-15) | ZKHP  (2016) | **Total** |  | TSDPS  (2015-18) |  |  |  |
|  |  |  |  |  |  |  |  |  |  | **≤10** | **>10** |
| **Individuals** |  | 64 | 11 | 32 | 13 | **88** |  | 88 |  |  |  |
| **Timepoints attended** |  |  |  |  |  |  |  |  |  |  |  |
| Minimum |  | 1 | 4 | 4 | 1 |  |  | 1 |  |  |  |
| Maximum |  | 2 | 4 | 4 | 1 |  |  | 1 |  |  |  |
| **Observations** |  |  |  |  |  |  |  |  |  |  |  |
| Dementia/CIND status |  |  |  |  |  |  |  | 88 |  |  |  |
| Education (highest) |  |  |  |  |  |  |  | 83 |  |  |  |
| Age (years) |  | 102 | 44 | 126 | 13 | **285** |  | 88 |  |  |  |
| Gender |  | 102 | 44 | 128 | 13 | **287** |  | 88 |  |  |  |
| Weight (kg) |  | 102 | 34 | 117 | 13 | **266** |  |  |  | 174 | 90 |
| BMI |  | 102 | 25 | 101 | 13 | **241** |  |  |  | 121 | 90 |
| Waist (cm) |  | 101 | 22 | 69 | 12 | **204** |  |  |  | 114 | 90 |
| Smoking |  | 102 | 42 | 87 | 13 | **244** |  |  |  | 109 | 90 |
| Alcohol |  | 102 | 38 | - | 13 | **153** |  |  |  | 62 | 90 |
| Systolic BP (mmHg) |  | 102 | 42 | 118 | 12 | **274** |  |  |  | 184 | 90 |
| Diastolic BP (mmHg) |  | 102 | 42 | 118 | 12 | **274** |  |  |  | 184 | 90 |
| Diabetes mellitus |  | 102 | 44 | 120 | 13 | **279** |  |  |  | 25 | 182 |
| Hypertension |  | 102 | 44 | 116 | 12 | **274** |  |  |  | 32 | 142 |
| HbA1c (% NGSP) |  | 43 | 44 | 118 | 11 | **216** |  |  |  | 185 | 31 |
| UACR (ratio) |  | 90 | 34 | 61 | 9 | **194** |  |  |  | 114 | 80 |
| Triglycerides (mmol/L) |  | 100 | 39 | 86 | 12 | **237** |  |  |  | 149 | 88 |
| Cholesterol (mmol/L) |  | 100 | 39 | 112 | 12 | **263** |  |  |  | 175 | 88 |
| HDL (mmol/L) |  | 100 | 39 | 112 | 12 | **263** |  |  |  | 175 | 88 |
| LDL (mmol/L) |  | 89 | 39 | 112 | 12 | **252** |  |  |  | 172 | 80 |
| VLDL (mmol/L) |  | 89 | - | - | 12 | **101** |  |  |  | 21 | 80 |

**Notes:** ‘-‘ = missing observations, CIND=Cognitive Impairment No Dementia, Weight=body weight (kilograms, kg), BMI=Body Mass Index [weight(kg)/height2], Waist=waist circumference (centimetres, cm), Smoking= self-reported “currently smoked tobacco,” Alcohol=self-reported any alcohol consumption (Yes/No), Systolic BP=systolic blood pressure, Diastolic BP=diastolic blood pressure, HbA1c=haemoglobin A1c (%), UACR=Urinary albumin creatinine ratio, HDL=high-density lipoprotein, LDL=low-density lipoprotein, VLDL=very low-density lipoprotein. Date information was missing for measures of hypertension (n=100), diabetes (n=72), smoking (n=45), BMI (n=30), and alcohol (n=1), so these variables have missing observations for the two time periods ≤10, >10 years before follow-up.

**Supplementary Table 2 – Number of winsorized observations across the Well Person’s Health Check (WPHC), the Getting Better at Chronic Care (GBACC) project, the Primary Health Care Models (PHCM) project and the Zenadth-Kes Health Partnership (ZKHP)**

| **Variables** | **WPHC  (1998-99) (2001-02)** | **GBACC  (2010-15)** | **PHCM  (2012-15)** | **ZKHP  (2016)** | **Total** |
| --- | --- | --- | --- | --- | --- |
|  |  |  |  |  |  |
| Weight | 1 | 2 | 4 | 0 | **7** |
| BMI | 1 | 1 | 3 | 0 | **5** |
| Waist | 3 | 1 | 1 | 0 | **5** |
| Systolic BP | 2 | 1 | 0 | 0 | **3** |
| Diastolic BP | 1 | 1 | 2 | 2 | **6** |
| HbA1c | 0 | 1 | 0 | 0 | **1** |
| UACR | 7 | 12 | 9 | 1 | **29** |
| Triglycerides | 7 | 0 | 2 | 0 | **9** |
| Cholesterol | 7 | 0 | 1 | 0 | **8** |
| HDL | 2 | 0 | 3 | 2 | **7** |
| LDL | 5 | 0 | 0 | 0 | **5** |
| VLDL | 0 | 0 | 0 | 0 | **0** |

**Notes:** Weight=body weight (kilograms, kg), BMI=Body Mass Index [weight(kg)/height2], Waist=waist circumference (centimetres, cm), Systolic BP=systolic blood pressure, Diastolic BP=diastolic blood pressure, HbA1c=haemoglobin A1c (%), UACR=Urinary albumin creatinine ratio, HDL=high-density lipoprotein, LDL=low-density lipoprotein, VLDL=very low-density lipoprotein.

**Supplementary Table 3 – Years between baseline measures from linked datasets and follow-up of individuals who participated in the Torres Strait Dementia Prevalence Survey (TSDPS)**

| **Variables** | **n** | **Mean** | **(sd)** | **Median** | **(iqr)** | **[Min-Max]** |
| --- | --- | --- | --- | --- | --- | --- |
| Smoking | 199 | 9.6 | 6.0 | 9 | (5 - 16) | [0 - 19] |
| Alcohol | 152 | 11.2 | 5.9 | 11 | (7 - 17) | [0 - 19] |
| Diabetes mellitus | 207 | 24.1 | 17.2 | 17 | (13 - 50) | [0 - 53] |
| Hypertension | 174 | 14.3 | 6.7 | 16 | (11 - 18) | [0 - 43] |
| Weight (kg) | 264 | 7.6 | 6.1 | 5 | (3 - 12) | [0 - 19] |
| BMI | 211 | 8.8 | 6.3 | 6 | (3 - 16) | [0 - 19] |
| Waist (cm) | 204 | 9.0 | 6.3 | 6 | (4 - 16) | [0 - 19] |
| Systolic BP (mmHg) | 274 | 7.5 | 6.0 | 5 | (3 - 12) | [0 - 19] |
| Diastolic BP (mmHg) | 274 | 7.5 | 6.0 | 5 | (3 - 12) | [0 - 19] |
| HbA1c (% NGSP) | 216 | 5.0 | 3.3 | 4 | (3 - 6) | [0 - 13] |
| UACR (ratio) | 194 | 8.8 | 6.1 | 6 | (4 - 15) | [0 - 19] |
| Triglycerides (mmol/L) | 237 | 8.3 | 6.1 | 5 | (4 - 13) | [0 - 19] |
| Cholesterol (mmol/L) | 263 | 7.7 | 6.0 | 5 | (3 - 12) | [0 - 19] |
| HDL (mmol/L) | 263 | 7.7 | 6.0 | 5 | (3 - 12) | [0 - 19] |
| LDL (mmol/L) | 252 | 7.4 | 6.0 | 5 | (3 - 12) | [0 - 19] |
| VLDL (mmol/L) | 101 | 13.0 | 5.8 | 15 | (10 - 17) | [0 - 19] |

**Notes:** CIND=Cognitive Impairment No Dementia, Weight=body weight (kilograms, kg), BMI=Body Mass Index [weight(kg)/height2], Waist=waist circumference (centimeters, cm), Smoking= self-reported “currently smoked tobacco,” Alcohol=self-reported any alcohol consumption (Yes/No), Systolic BP=systolic blood pressure, Diastolic BP=diastolic blood pressure, HbA1c=hemoglobin A1c (%), UACR=Urinary albumin creatinine ratio, HDL=high-density lipoprotein, LDL=low-density lipoprotein, VLDL=very low-density lipoprotein.

**Supplementary Table 4 – Completeness of variables by time of measurement across the Well Person’s Health Check (WPHC), the Getting Better at Chronic Care (GBACC) project, the Primary Health Care Models (PHCM) project, the Zenadth-Kes Health Partnership (ZKHP) project and the Torres Strait Dementia Prevalence Survey (TSDPS)**

|  |  | **Baseline assessments** | | | | |
| --- | --- | --- | --- | --- | --- | --- |
| **Variables** |  | WPHC  (1998-99) (2001-02) | GBACC  (2010-15) | PHCM  (2012-15) | ZKHP  (2016) | **Total** |
|  |  |  |  |  |  |  |
| **Observations** |  |  |  |  |  |  |
|  |  |  |  |  |  |  |
| **Within 10 years** |  |  |  |  |  |  |
| Weight (kg) |  | 12 | 34 | 115 | 13 | **174** |
| BMI |  | 12 | 25 | 71 | 13 | **121** |
| Waist (cm) |  | 11 | 22 | 69 | 12 | **114** |
| Smoking |  | 12 | 38 | 46 | 13 | **109** |
| Alcohol |  | 12 | 37 | - | 13 | **62** |
| Systolic BP (mmHg) |  | 12 | 42 | 118 | 12 | **184** |
| Diastolic BP (mmHg) |  | 12 | 42 | 118 | 12 | **184** |
| Diabetes mellitus |  | 12 | 0 | 0 | 13 | **25** |
| Hypertension |  | 12 | 4 | 4 | 12 | **32** |
| HbA1c (% NGSP) |  | 12 | 44 | 118 | 11 | **185** |
| UACR (ratio) |  | 10 | 34 | 61 | 9 | **114** |
| Triglycerides (mmol/L) |  | 12 | 39 | 86 | 12 | **149** |
| Cholesterol (mmol/L) |  | 12 | 39 | 112 | 12 | **175** |
| HDL (mmol/L) |  | 12 | 39 | 112 | 12 | **175** |
| LDL (mmol/L) |  | 9 | 39 | 112 | 12 | **172** |
| VLDL (mmol/L) |  | 9 | - | - | 12 | **21** |
| **After 10 years** |  |  |  |  |  |  |
| Weight (kg) |  | 90 | 0 | - | 0 | **90** |
| BMI |  | 90 | 0 | 0 | 0 | **90** |
| Waist (cm) |  | 90 | 0 | - | 0 | **90** |
| Smoking |  | 90 | 0 | 0 | 0 | **90** |
| Alcohol |  | 90 | 0 | - | 0 | **90** |
| Systolic BP (mmHg) |  | 90 | 0 | 0 | 0 | **90** |
| Diastolic BP (mmHg) |  | 90 | 0 | 0 | 0 | **90** |
| Diabetes mellitus |  | 90 | 40 | 52 | 0 | **182** |
| Hypertension |  | 90 | 18 | 34 | 0 | **142** |
| HbA1c (% NGSP) |  | 31 | 0 | 0 | 0 | **31** |
| UACR (ratio) |  | 80 | 0 | 0 | 0 | **80** |
| Triglycerides (mmol/L) |  | 88 | 0 | 0 | 0 | **88** |
| Cholesterol (mmol/L) |  | 88 | 0 | 0 | 0 | **88** |
| HDL (mmol/L) |  | 88 | 0 | 0 | 0 | **88** |
| LDL (mmol/L) |  | 80 | 0 | 0 | 0 | **80** |
| VLDL (mmol/L) |  | 80 | - | - | 0 | **80** |

**Notes:** ‘-‘ = missing observations, CIND=Cognitive Impairment No Dementia, Weight=body weight (kilograms, kg), BMI=Body Mass Index [weight(kg)/height2], Waist=waist circumference (centimeters, cm), Smoking= self-reported “currently smoked tobacco,” Alcohol=self-reported any alcohol consumption (Yes/No), Systolic BP=systolic blood pressure, Diastolic BP=diastolic blood pressure, HbA1c=hemoglobin A1c (%), UACR=Urinary albumin creatinine ratio, HDL=high-density lipoprotein, LDL=low-density lipoprotein, VLDL=very low-density lipoprotein.

**Supplementary Table 5 – Distribution of baseline variables from linked datasets of 88 individuals who participated in the Torres Strait Dementia Prevalence Survey (TSDPS) with at least one baseline assessment**

| **Variables** |  |  |  |  |
| --- | --- | --- | --- | --- |
|  |  |  |  |  |
| **Follow up measures** | **n** | **(%)** |  |  |
| **Total** | **88** | **(100.0)** |  |  |
| Diagnosis |  |  |  |  |
| Normal | 55 | (62.5) |  |  |
| CIND | 26 | (29.5) |  |  |
| Dementia | 7 | (8.0) |  |  |
| *CIND/Dementia* | *33* |  |  |  |
| Gender |  |  |  |  |
| Male | 24 | (27.3) |  |  |
| Female | 64 | (72.7) |  |  |
| Education (3 levels) |  |  |  |  |
| Primary | 27 | (32.5) |  |  |
| Any high school | 23 | (27.7) |  |  |
| Post school | 33 | (39.8) |  |  |
| Education (2 levels) |  |  |  |  |
| Grade school | 50 | (60.2) |  |  |
| Post school | 33 | (39.8) |  |  |
|  |  |  |  |  |
| **Baseline measures** | **n** | **(%)** |  |  |
| Smoking |  |  |  |  |
| No | 195 | (79.9) |  |  |
| Yes | 49 | (20.1) |  |  |
| Alcohol |  |  |  |  |
| No | 91 | (59.5) |  |  |
| Yes | 62 | (40.5) |  |  |
| Diabetes mellitus |  |  |  |  |
| No | 60 | (21.5) |  |  |
| Yes | 219 | (78.5) |  |  |
| Hypertension |  |  |  |  |
| No | 60 | (21.9) |  |  |
| Yes | 214 | (78.1) |  |  |
| Albuminuria |  |  |  |  |
| No | 113 | (58.2) |  |  |
| Yes | 81 | (41.8) |  |  |
| UACR (≥14) |  |  |  |  |
| No | 147 | (75.8) |  |  |
| Yes | 47 | (24.2) |  |  |
| Triglycerides (≤0.8 mmol/L) |  |  |  |  |
| No | 201 | (84.8) |  |  |
| Yes | 36 | (15.2) |  |  |
| Cholesterol (≤3.5 mmol/L) |  |  |  |  |
| No | 212 | (80.6) |  |  |
| Yes | 51 | (19.4) |  |  |
| LDL (≤1.7 mmol/L) |  |  |  |  |
| No | 213 | (84.5) |  |  |
| Yes | 39 | (15.5) |  |  |
|  |  |  |  |  |
| Continuous | n | Mean (sd) | Med (iqr) | [Min-Max] |
| Age (years) |  |  |  |  |
| TSDPS (2015-18) | 88 | 65.9 (10.4) | 65 (59-74) | [44-93] |
| ZKHP (2016) | 13 | 63.8 (7.4) | 63 (60-69) | [49-75] |
| PHCM (2012-15) | 126 | 65.1 (10.5) | 65 (57-74) | [41-92] |
| GBACC (2010-15) | 44 | 57.4 (5.5) | 56 (52-63) | [48-65] |
| WPHC (1998-99, 2001-02) | 102 | 51.7 (10.7) | 52 (44-59) | [25-75] |
|  |  |  |  |  |
| Weight (kg) | 266 | 91.4 (19.8) | 89 (79-103) | [49-141] |
| BMI | 241 | 33.6 (7.3) | 32 (29-38) | [18-52] |
| Waist (cm) | 204 | 112.0 (14.5) | 112 (103-121) | [82-149] |
| Systolic BP (mmHg) | 274 | 131.9 (16.7) | 130 (120-144) | [96-179] |
| Diastolic BP (mmHg) | 274 | 73.7 (11.6) | 74 (66-80) | [45-101] |
| HbA1c (% NGSP) | 216 | 8.1 (2.1) | 8 (6-9) | [5-14] |
| UACR (ratio) | 194 | 8.5 (11.9) | 2 (1-15) | [0-41] |
| Triglycerides (mmol/L) | 237 | 1.7 (0.8) | 2 (1-2) | [1-4] |
| Cholesterol (mmol/L) | 263 | 4.5 (1.0) | 4 (4-5) | [3-7] |
| HDL (mmol/L) | 263 | 1.1 (0.2) | 1 (1-1) | [1-2] |
| LDL (mmol/L) | 252 | 2.7 (0.9) | 3 (2-3) | [0-5] |
| VLDL (mmol/L) | 101 | 0.8 (0.4) | 1 (1-1) | [0-2] |

**Notes:** CIND=Cognitive Impairment No Dementia, Weight=body weight (kilograms, kg), BMI=Body Mass Index [weight(kg)/height2], Waist=waist circumference (centimeters, cm), Smoking= self-reported “currently smoked tobacco,” Alcohol=self-reported any alcohol consumption (Yes/No), Systolic BP=systolic blood pressure, Diastolic BP=diastolic blood pressure, HbA1c=hemoglobin A1c (%), UACR=Urinary albumin creatinine ratio, HDL=high-density lipoprotein, LDL=low-density lipoprotein, VLDL=very low-density lipoprotein. WPHC=Well Person’s Health Check, GBACC=Getting Better at Chronic Care, PHCM=Primary Health Care Models, ZKHP=Zenadth-Kes Health Partnership.

**Supplementary Table 6 – Distribution of winsorized baseline variables by timing of collection and CIND/dementia status at follow-up for 88 individuals who participated in the Torres Strait Dementia Prevalence Survey (TSDPS) and at least one baseline assessment**

| **Variables** |  | **Normal** | |  |  | **CIND/Dementia** | |  | **Tests** | |
| --- | --- | --- | --- | --- | --- | --- | --- | --- | --- | --- |
|  |  | **n** | **(%)** |  |  | **n** | **(%)** |  |  |  |
| **Follow up (Individuals)** |  | **55** | **(100.0)** |  |  | **33** | **(100.0)** |  |  |  |
|  |  |  |  |  |  |  |  |  |  |  |
| **Baseline (Observations)** |  |  | |  |  |  | |  |  |  |
| **Within 10 years** |  |  |  |  |  |  |  |  |  |  |
|  | n | Mean (sd) / Med (iqr) | |  | n | Mean (sd) / Med (iqr) | |  | RR | p |
| Weight (kg)^1^ | 101 | 96.4 | (21.4) |  | 73 | 84.8 | (16.9) |  | 0.98 | <0.001 |
| HbA1c (% NGSP)^1^ | 112 | 8.3 | (2.2) |  | 73 | 8.1 | (2.1) |  | 0.97 | 0.544 |
| UACR (ratio)^2^ | 62 | 1.7 | (0.6-20.0) |  | 52 | 2.8 | (1.0-29.0) |  | 5.55 | 0.019 |
| Triglycerides (mmol/L)^2^ | 88 | 1.7 | (1.1-2.2) |  | 61 | 1.4 | (1.0-1.8) |  | 5.48 | 0.019 |
|  |  |  |  |  |  |  |  |  |  |  |
| **After 10 years** |  |  |  |  |  |  |  |  |  |  |
| Weight (kg)^1^ | 64 | 94.5 | (19.3) |  | 26 | 82.8 | (15.2) |  | 0.97 | 0.001 |
| UACR (ratio)^2^ | 56 | 1.1 | (0.6-5.3) |  | 24 | 2.4 | (0.6-9.6) |  | 0.82 | 0.364 |
| VLDL (mmol/L)^2^ | 59 | 0.8 | (0.5-1.1) |  | 21 | 0.7 | (0.4-0.8) |  | 3.20 | 0.074 |

**Notes:** Within 10 years≤0.0-10.0 years, After 10 years>10.0 years, Weight=body weight (kilograms, kg), HbA1c=haemoglobin A1c (%), UACR=Urinary albumin creatinine ratio, VLDL=very low-density lipoprotein. 1=Normally distributed, differences tested with Independent samples t-test, 2=Not normally distributed, differences tested with Kruskal-Wallis rank sum tests)

**Supplementary Table 7 – Mixed Effects Generalized Linear Modelling (meglm) with relative risk (RR) associations between selected winsorized baseline measures and a diagnosis of Cognitive Impairment No Dementia (CIND) or dementia at follow-up for 88 people who participated in at least one baseline study, unadjusted and adjusted for age and years between baseline measure and follow-up assessment**

| **Variables** |  | **CIND or Dementia** |  | **Adjusted - Age** | | |  | **Adjusted – Age & Years** | | |
| --- | --- | --- | --- | --- | --- | --- | --- | --- | --- | --- |
|  | **N** | **No / Yes** |  | **RR** | **(95%CI)** | **p** |  | **RR** | **(95%CI)** | **p** |
| Within 10 years |  |  |  |  |  |  |  |  |  |  |
| Weight (kg) | 174 | 101 / 73 |  | 0.99 | (0.97-1.01) | 0.294 |  | 0.99 | (0.97-1.01) | 0.296 |
| UACR (ratio) | 114 | 62 / 52 |  | 1.02 | (1.01-1.04) | 0.000 |  | 1.02 | (1.01-1.04) | 0.000 |
| Triglycerides (mmol/L) | 149 | 88 / 61 |  | 0.75 | (0.49-1.14) | 0.182 |  | 0.75 | (0.49-1.14) | 0.186 |
|  |  |  |  |  |  |  |  |  |  |  |
| After 10 years |  |  |  |  |  |  |  |  |  |  |
| Weight (kg) | 90 | 64 / 26 |  | 0.98 | (0.95-1.00) | 0.074 |  | 0.98 | (0.95-1.00) | 0.056 |
| UACR (ratio) | 80 | 56 / 24 |  | 1.02 | (1.00-1.04) | 0.019 |  | 1.02 | (1.00-1.04) | 0.061 |
| VLDL (mmol/L) | 80 | 59 / 21 |  | 0.27 | (0.07-1.03) | 0.056 |  | 0.31 | (0.09-1.02) | 0.053 |

**Supplementary Table 8 – Main analyses excluding ZKHP participants (n=13), Mixed Effects Generalized Linear Modelling (meglm) with relative risk (RR) associations between selected baseline measures and a diagnosis of Cognitive Impairment No Dementia (CIND) or dementia at follow-up for 88 people who participated in at least one baseline study, adjusted for age and years between measure and follow-up**

| **Variables** |  |  | **Adjusted - Age, Years** | | |
| --- | --- | --- | --- | --- | --- |
|  | **N** |  | **RR** | **(95%CI)** | **p** |
|  |  |  |  |  |  |
| Original measures |  |  |  |  |  |
| Weight (kg) | 251 |  | 0.98 | (0.97-1.00) | 0.062 |
| HbA1c (% NGSP) | 205 |  | 1.06 | (0.96-1.17) | 0.248 |
| UACR (ratio) | 185 |  | 1.00 | (1.00-1.00) | 0.551 |
| Triglycerides (mmol/L) | 225 |  | 0.91 | (0.71-1.17) | 0.467 |
| Cholesterol (mmol/L) | 251 |  | 0.86 | (0.70-1.07) | 0.172 |
| VLDL (mmol/L) | 89 |  | 0.33 | (0.10-1.09) | 0.070 |
|  |  |  |  |  |  |
| Winzorised outliers |  |  |  |  |  |
| Weight (kg) | 251 |  | 0.98 | (0.97-1.00) | 0.070 |
| HbA1c (% NGSP) | 205 |  | 1.06 | (0.96-1.17) | 0.239 |
| UACR (ratio) | 185 |  | 1.02 | (1.01-1.03) | 0.000 |
| Triglycerides (mmol/L) | 225 |  | 0.76 | (0.55-1.05) | 0.099 |
| Cholesterol (mmol/L) | 251 |  | 0.86 | (0.69-1.07) | 0.171 |
|  |  |  |  |  |  |
| Outliers removed |  |  |  |  |  |
| Weight (kg) | 244 |  | 0.99 | (0.97-1.00) | 0.118 |
| HbA1c (% NGSP) | 204 |  | 1.06 | (0.96-1.17) | 0.219 |
| UACR (ratio) (n=29) | 157 |  | 1.02 | (1.01-1.03) | 0.000 |
| UACR (ratio) (n=2) | 183 |  | 1.00 | (1.00-1.01) | 0.005 |
| Triglycerides (mmol/L) | 216 |  | 0.68 | (0.47-0.99) | 0.045 |
| Cholesterol (mmol/L) | 243 |  | 0.84 | (0.67-1.06) | 0.151 |

**Supplementary Files**

Supplementary File 1 – Example of individual observations for triglycerides and anonymised example structure of the dataset used data for analyses

Supplementary File 2 – Baseline pathology measures before and after winsorizing, by follow-up dementia status, for 88 individuals who participated in the Dementia Prevalence Survey and had at least one baseline assessment
